# Nonlinear causal effects of estimated glomerular filtration rate on myocardial infarction risks: Mendelian randomization study

**DOI:** 10.1186/s12916-022-02251-1

**Published:** 2022-02-03

**Authors:** Sehoon Park, Soojin Lee, Yaerim Kim, Semin Cho, Hyeok Huh, Kwangsoo Kim, Yong Chul Kim, Seung Seok Han, Hajeong Lee, Jung Pyo Lee, Kwon Wook Joo, Chun Soo Lim, Yon Su Kim, Dong Ki Kim

**Affiliations:** 1 Department of Biomedical Sciences, Seoul National University College of Medicine, Seoul, South Korea; 2 Department of Internal Medicine, Armed Forces Capital Hospital, Gyeonggi-do, South Korea; 3 Department of Internal Medicine, Uijeongbu Eulji University Medical Center, Seoul, South Korea; 4 Department of Internal Medicine, Seoul National University College of Medicine, Seoul, South Korea; 5 Department of Internal Medicine, Keimyung University School of Medicine, Daegu, South Korea; 6 Department of Internal Medicine, Seoul National University Hospital, Seoul, South Korea; 7 Transdisciplinary Department of Medicine & Advanced Technology, Seoul National University Hospital, Seoul, South Korea; 8 Kidney Research Institute, Seoul National University, Seoul, South Korea; 9 Department of Internal Medicine, Seoul National University Boramae Medical Center, Seoul, South Korea

**Keywords:** Myocardial infarction, Kidney, Mendelian randomization

## Abstract

**Background:**

Previous observational studies suggested that a reduction in estimated glomerular filtration rate (eGFR) or a supranormal eGFR value was associated with adverse cardiovascular risks. However, a previous Mendelian randomization (MR) study under the linearity assumption reported null causal effects from eGFR on myocardial infarction (MI) risks. Further investigation of the nonlinear causal effect of kidney function assessed by eGFR on the risk of MI by nonlinear MR analysis is warranted.

**Methods:**

In this MR study, genetic instruments for log-eGFR based on serum creatinine were developed from European samples included in the CKDGen genome-wide association study (GWAS) meta-analysis (*N*=567,460). Alternate instruments for log-eGFR based on cystatin C were developed from a GWAS of European individuals that included the CKDGen and UK Biobank data (*N*=460,826). Nonlinear MR analysis for the risk of MI was performed using the fractional polynomial method and the piecewise linear method on data from individuals of white British ancestry in the UK Biobank (*N*=321,024, with 12,205 MI cases).

**Results:**

Nonlinear MR analysis demonstrated a U-shaped (quadratic *P* value < 0.001) association between MI risk and genetically predicted eGFR (creatinine) values, as MI risk increased as eGFR declined in the low eGFR range and the risk increased as eGFR increased in the high eGFR range. The results were similar even after adjustment for clinical covariates, such as blood pressure, diabetes mellitus, dyslipidemia, or urine microalbumin levels, or when genetically predicted eGFR (cystatin C) was included as the exposure.

**Conclusion:**

Genetically predicted eGFR is significantly associated with the risk of MI with a parabolic shape, suggesting that kidney function impairment, either by reduced or supranormal eGFR, may be causally linked to a higher MI risk.

**Supplementary Information:**

The online version contains supplementary material available at 10.1186/s12916-022-02251-1.

## Background

The kidney is a vital organ for volume homeostasis, uremic toxin clearance, maintenance of body electrolyte balance, and various biological functions. A state of impaired kidney function, chronic kidney disease (CKD), is an emerging comorbidity with a high prevalence and large socioeconomic burden, thus, assessment of kidney function with the estimated glomerular function rate (eGFR) is commonly performed in diverse clinical conditions [1].

The close linkage between myocardial infarction (MI) and eGFR has been noted previously [2, 3]. CKD is one of the most widely acknowledged risk factors for MI, and reduced eGFR is associated with a poor prognosis in MI patients. In addition, recent observational studies reported that a state of supranormal eGFR, kidney hyperfiltration, was associated with a higher risk of cardiovascular diseases [4–7]. To further confirm that the observational findings were from the causal effects on eGFR on MI risks, Mendelian randomization (MR) analysis, an analytical tool widely used in the recent medical literature, can be helpful. MR analysis has strengths in demonstrating causal estimates minimally affected by reverse causation or confounding effects, as the method implements inborn-fixed genetic instrument variables. However, previous MR studies reported null causal effects of kidney function parameters on MI [8, 9], a finding that was contradictory to previous observational findings. Nevertheless, considering that a causal effect may be nonlinear, conventional summary-level MR analysis would not capture the complex exposure-outcome relation because it assumes linearity, particularly for a suspected U-shaped relationship between eGFR and cardiovascular risk [7, 10, 11]. Therefore, further nonlinear MR analysis is warranted to investigate the shape of the causal estimates on MI according to eGFR values.

In this study, we hypothesized that a causal effect of eGFR on MI risk would be present with a nonlinear exposure-outcome relationship. We performed a nonlinear MR analysis utilizing the largest individual-level genetic database that includes MI phenotyping and eGFR measurements, the UK Biobank.

## Methods

### Ethical considerations

The study was performed in accordance with the Declaration of Helsinki and approved by the Institutional Review Boards of Seoul National University Hospital (No. E-2006-043-1131). The usage of the UK Biobank data was approved by the UK Biobank consortium (application No. 53799). Acquisition of informed consent was not required, as the study investigated anonymous public databases and genetic summary statistics.

### Study setting

**Fig. 1:**
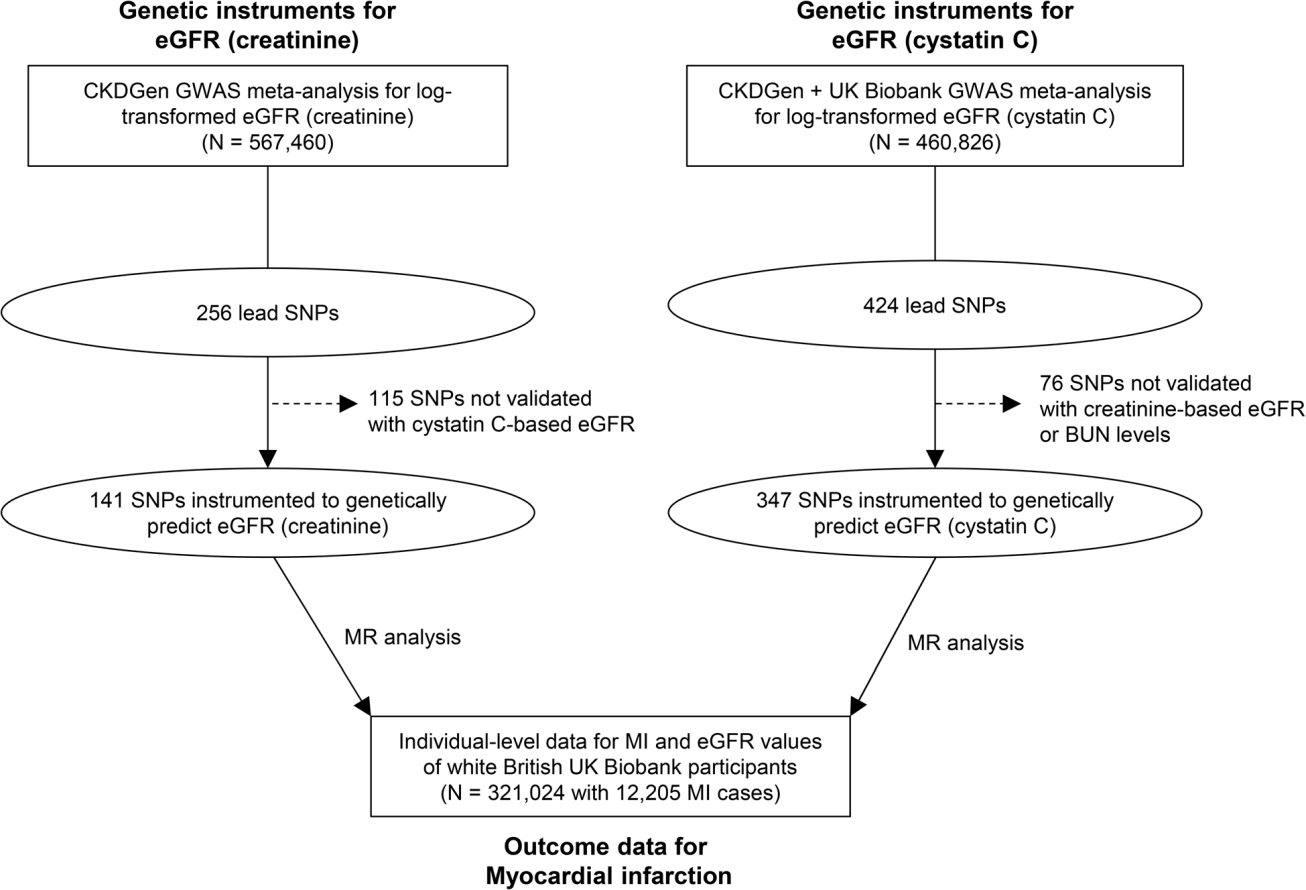
Study flow diagram. eGFR, estimated glomerular filtration rate; GWAS, genome-wide association study; SNP, single-nucleotide polymorphism; BUN, blood urea nitrogen; MR, Mendelian randomization

The study was an MR analysis of the major findings from the CKDGen and UK Biobank data (Fig. 1). The CKDGen genome-wide association study (GWAS) meta-analysis provides the largest-scale information to date on single-nucleotide polymorphisms (SNPs) associated with kidney function traits (URL: https://ckdgen.imbi.uni-freiburg.de/) [12, 13], thus, it was utilized to develop the genetic instruments for log-transformed eGFR. The UK Biobank is a population-scale prospective cohort that included > 500,000 participants aged 40–69 from diverse regions in the UK from 2006 to 2010 (URL: https://www.ukbiobank.ac.uk/) [14]. The database includes a variety of clinicodemographic information and deep genotyping data and thus has been widely used for genetic studies, including MR analysis.

The main analysis was a two-sample MR analysis; genetic instruments were developed for log-transformed eGFR values calculated by the CKD-EPI equation using creatinine values from the phase 4 CKDGen GWAS meta-analysis [12], and MI outcomes in the UK Biobank with no sample overlap between the two data. Avoiding sample overlap in MR analysis has strength in a conservative sense, as a potential bias, particularly in the case of weak instruments, is toward false negative findings; thus, the robustness of a positive finding by two-sample MR analysis can be supported [15].

We also performed a secondary analysis using genetic instruments for log-transformed eGFR values based on serum cystatin C levels [13], as creatinine-based eGFR values are more likely to be biased by dietary factors or body shapes than cystatin C-based levels. Genetic instruments were developed from a recent GWAS meta-analysis that included both CKDGen studies and UK Biobank data. As the UK Biobank provided > 90% of the samples for the meta-analysis, the MR analysis is nearly a one-sample setting. Despite the sample overlap-related issues, the analysis has strength for replicative purposes, including an alternate kidney function parameter which is less affected from external factors.

### MR assumptions

Three core assumptions should be attained to demonstrate causal estimates by an MR analysis [16]. First, the relevance assumption is that the genetic instrument should be closely associated with the exposure phenotype, and as the instruments were associated with eGFR with genome-wide significance, this assumption was considered attained. We further tested the association strength by calculating the explained variance by R2 values. The other two assumptions, the independence and the exclusion-restriction assumptions, are regarded as an absence of a pleiotropic pathway. The independence assumption means that an instrument should not be associated with a confounder, and the exclusion-restriction assumption means that the causal effect should occur through the exposure of interest. In MR analysis under the linearity assumption, some pleiotropy-robust MR analyses are usually performed to address and test the pleiotropic effects (e.g., MR-Egger regression). Alternatively, in the current study, non-linear MR analysis directly adjusting the potential confounding covariates was performed to conduct a MR analysis even controlling some measured pleiotropic effects. In addition, we trimmed the genetic instruments to exclude the variants that are suspected to be weakly associated with kidney function traits because of potential confounding associations.

### Genetic instruments for log-transformed eGFR based on creatinine levels

For the genetic instruments in the main two-sample MR analysis, we used the results of individuals of European ancestry (*N*=567,460) from the CKDGen data [12] to restrict our analysis to individuals of a single ancestry, as in our previous studies [17–19]. The European participants had a median age of 54 years old, 91.4 mL/min/1.73 m^2^ median eGFR values, and 9% prevalence of CKD determined by eGFR < 60 mL/min/1.73 m^2^, and 50% of them were male.

The CKDGen GWAS meta-analysis reported 256 index SNPs at least 1 Mbp apart with a genome-wide significant association (*P* < 5 × 10^−8^) with log-transformed eGFR values based on creatinine levels. Additional trimming of the 256 SNPs was necessary, as in our previous MR analyses [17–19], to remove genetic variants likely related to creatinine metabolism instead of kidney function. We performed an association analysis of eGFR based on cystatin C levels with data from 337,138 individuals of white British ancestry in the UK Biobank who passed genetic quality control with the exclusion of those outliers for heterozygosity, missing rate, or with sex chromosome aneuploidy. The variables for genetic quality control were predefined by the UK Biobank consortium, and those who were included in the principal component analysis and those who reported white British ancestry were included. The linear regression analysis of the 256 SNPs for the cystatin C eGFR level was adjusted for age, sex, age × sex, age^2^, and the first 10 genetic principal components by PLINK 2.0 [20]. Other details of the genetic data structure and quality control process are available in the resources provided by the UK Biobank consortium [14]. We disregarded 115 SNPs identified from the association analysis in the UK Biobank data that showed different directions of regressed betas or those not reaching the Bonferroni adjusted significance level (*P* < 0.05/256) association with cystatin C-based eGFR values. Finally, the remaining 141 SNPs and their summary statistics in the CKDGen data were used as the genetic instrument for kidney function, and the combined allele score explained 2.69% of the variance in creatinine-based eGFR values in the UK Biobank data. When we calculated the F statistic, which should be over 10 to avoid weak instrument bias, by the equation [(*n* – *k* − 1)/(*k*)]*[*R*2/(1 − *R*2)], where *n* represents sample size, *k* represents the number of instruments, and *R*2 represents explained variance of the exposure phenotype, the F statistic was 66.1 [17]. The summary statistics for the instrumented SNPs are presented in Supplemental Table 1.

### Genetic instruments for log-transformed eGFR based on cystatin C levels

As serum creatinine levels are affected by muscle mass or diet, the cystatin C-based eGFR value has certain benefits when assessing kidney function [21]. Cystatin C-based eGFR value was a superior biomarker in regard to its power to predict cardiovascular diseases in the UK Biobank data [22].

A recent GWAS meta-analysis incorporating CKDGen studies and UK Biobank data reported 424 lead SNPs with genome-wide significant association with log-transformed eGFR based on creatinine levels. The study included a GWAS meta-analysis for log-transformed eGFR values based on cystatin C levels of samples from individuals of European ancestry (*N*=460,826) [13]. To include the SNPs consistently associated with kidney function-related biomarker, a trimming was performed similar to the aforementioned method and 348 SNPs were validated to be significantly (*P* < 0.05) associated with eGFR based on creatinine and blood urea nitrogen levels with consistent direction. We used the information of these 348 SNPs and the statistics of their association with eGFR based on cystatin C levels as the genetic instruments for the secondary analysis (Supplemental Table 2). Combined allele scores of the 348 SNPs explained 3.47% of the variance in cystatin C-based eGFR values in the data from individuals of white British ancestry in the UK Biobank, yielding an F statistic of 34.8 [23].

### MI outcome in the UK Biobank data

We used the UK Biobank data as the source of MI outcome, as individual-level large-scale genetic data, including information on both eGFR values and MI events, are necessary for a nonlinear MR analysis [10, 17]. The UK Biobank data defined MI events based on self-reports, hospital admission records, and death registries throughout the UK. Among the 337,138 individuals of white British ancestry in the UK Biobank data used in this study, 321,024 individuals had available creatinine- and cystatin C-based eGFR values, including 12,205 people with MI.

### Nonlinear MR analysis

Conventional MR analysis (e.g., inverse variance weighted method) assumes a linear exposure-outcome relationship. As average causal estimates are calculated for the total ranges of an exposure in such an analysis, the causal estimates can be falsely attenuated if the true exposure-outcome relationship is nonlinear [10]. In such conditions, nonlinear MR analysis can be used.

In nonlinear MR analysis, the stratification of the population is performed by instrument-free exposure, the residual variation in the exposure conditioned for the instruments [10]. This is because directly dividing the study population according to exposure phenotype would bias the results by invalidating the MR assumptions; thus, the nongenetic component of the exposure is used to stratify the population. Next, localized averaged causal estimates are calculated as the association between the outcome and genetically predicted exposure divided by the association between the exposure and genetically predicted exposure. Finally, meta-regression of the localized causal estimates can be performed in nonlinear MR analysis to estimate the exposure-outcome relationship.

First, we plotted restricted cubic spline curves from the instrument-free exposure on MI risks to present interpretable MR estimates. The cubic spline curves based on logistic regression analysis for MI outcome was plotted with 10 knots determined based on decile values.

For non-linear MR analysis, we mainly used the fractional polynomial model [10], one of the methods that have been commonly used in recent non-linear MR studies [24, 25]. The degree 1 or degree 2 model is commonly used for nonlinear MR analysis, and whether the degree 2 model fits better, particularly when the exposure-outcome association is complex, can be tested. In the current study, we used the flexible degree 2 model, as the model fit was better (*P* = 0.004) than that of the degree 1 model, for the main two-sample MR analysis with 100 strata [10]. Allele scores for genetically predicted eGFR were calculated with PLINK 2.0 by multiplying the gene dosage matrix with the regressed betas from the GWAS summary statistics, which provided the genetic instruments [20]. Whether the scores followed a normal distribution was assessed by histograms, as a normal distribution supports the random allocation of genotypes, which is necessary for MR analysis [26]. The main nonlinear MR analysis included adjustments for the covariates age, sex, and the first 10 genetic principal components. The risks of MI according to creatinine-based eGFR or cystatin C-based eGFR, calculated by the CKD-EPI equation [27, 28], were investigated by nonlinear MR analysis. To robustly control the effects from clinical covariables, we additionally adjusted for body mass index, systolic blood pressure, hypertension medication history, hemoglobin A1c, history of diabetes diagnosis, levels of triglycerides, high-density lipoprotein, low-density lipoprotein, dyslipidemia medication history, and urine microalbumin levels in a sensitivity analysis (Supplemental Methods). The sensitivity analysis was performed on 245,398 individuals (9128 MI patients) with complete information on the covariates.

We additionally presented the results by piecewise linear method from the same models constructed in the above analysis by the fractional polynomial method.

The nonlinear MR analysis was performed by the “nlmr” package in R [10], and a two-sided *P* value < 0.05 was considered a significant finding. The reference point of the phenotypical eGFR value for the analysis was designated as 90 mL/min/1.73 m^2^, which was suggested by the clinical guideline and was reported to be associated with minimal cardiovascular risks in previous observational studies [7].

### Conventional summary-level MR analysis

We performed supplemental summary-level MR analysis by the inverse variance weighted method, weighted median method [29], and MR-Egger regression [30] to inspect causal estimates under the linearity assumption [31]. The analysis was first performed against the outcome data from individuals of white British ancestry in the UK Biobank, and the summary statistics for MI risk were generated by a GWAS adjusted for age, sex, age × sex, age^2^, and the first 10 genetic principal components by PLINK 2.0 [20]. A replicative analysis was performed on the summary statistics provided by the CARDIoGRAMplusC4D consortium, which was from a GWAS meta-analysis including 43,676 MI cases and 128,199 controls of predominantly European ancestry samples who were not included in the UK Biobank data [32]. The other details for the summary-level MR analysis are presented in the Supplemental Methods.

## Results

### Characteristics of the UK Biobank outcome data

At the baseline visits, the median age of the 321,024 individuals of white British ancestry was 58 years, and 46% of them were male (Table 1). The median creatinine-based eGFR and cystatin C-based eGFR values were 92.50 (2.3% with < 60) and 88.89 (4.7% with < 60) mL/min/1.73 m^2^, respectively (Supplemental Fig. 1). Four percent (13,205 cases) had prevalent/incident MI events, and the proportion was higher in males (7%) than in females (2%).

**Table 1:** Characteristics of the outcome dataset of individuals of white British ancestry in the UK Biobank

	Total	Female	Male
*N*	(*N*=321,024)	(*N*=172,289)	(*N*=148,735)
Age (years)	58 [51;63]	58 [50;63]	59 [51;64]
Sex			
Female	172,289 (54%)	172,289 (100.00%)	0 (0%)
Male	148,735 (46%)	0 (0.0%)	148,735 (100%)
Body mass index (kg/m^2^)	26.7 [24.1;29.8]	26.1 [23.4;29.6]	27.3 [25.0;30.0]
Obesity (> 30 kg/m^2^)	77,051 (24%)	39,635 (23%)	37,416 (25%)
Hypertension medication	66,676 (21%)	29,946 (17%)	36,730 (25%)
Systolic BP (mmHg)	136.5 [125;149.5]	133.5 [121.5;147.5]	139.5 [129;152]
Diastolic BP (mmHg)	82 [75.5;89]	80 [73.5;87]	84 [77.5;90.5]
Diabetes mellitus	15,368 (5%)	5830 (3%)	9538 (6%)
Hemoglobin A1c (mmol/L)	35.1 [32.7;37.7]	35.1 [32.7;37.6]	35.2 [32.7;37.9]
Dyslipidemia medication	55,731 (17%)	21,609 (13%)	34,122 (23%)
Triglycerides (mmol/L)	1.49 [1.05;2.16]	1.34 [0.97;1.90]	1.70 [1.19;2.45]
LDL cholesterol (mmol/L)	3.53 [2.96;4.13]	3.59 [3.02;4.20]	3.47 [2.88;4.06]
HDL cholesterol (mmol/L)	1.40 [1.18;1.68]	1.56 [1.33;1.83]	1.24 [1.07;1.46]
eGFR (creatinine, mL/min/1.73 m^2^)	92.50 [82.61;99.54]	92.86 [82.59;99.79]	92.16 [82.62;99.25]
< 30	301 (0.1%)	127 (0.1%)	174 (0.1%)
≥ 30 and < 60	7063 (2.2%)	3781 (2.2%)	3282 (2.2%)
≥ 60 and < 90	126,376 (39.4%)	66,615 (38.7%)	59,761 (40.2%)
≥ 90 and < 120	186,747 (58.2%)	101,584 (59.0%)	85,163 (57.3%)
≥ 120	537 (0.2%)	182 (0.1%)	355 (0.2%)
eGFR (cystatin C, mL/min/1.73 m^2^)	88.89 [77.13;100.48]	89.85 [77.48;100.92]	87.88 [76.72;99.73]
< 30	519 (0.2%)	214 (0.1%)	305 (0.2%)
≥ 30 and < 60	14,365 (4.5%)	7602 (4.4%)	6763 (4.6%)
≥ 60 and < 90	153,403 (47.8%)	78,858 (45.8%)	74,545 (50.1%)
≥ 90 and < 120	151,157 (47.1%)	84,976 (49.3%)	66,181 (44.5%)
≥ 120	1580 (0.5%)	639 (0.4%)	941 (0.6%)
Myocardial infarction	13,205 (4%)	3111 (2%)	10,094 (7%)

Continuous values are presented as medians [interquartile ranges], and categorical values are presented as *N* (%)

*BP* blood pressure, *LDL* low-density lipoprotein, *HDL* high-density lipoprotein, *eGFR* estimated glomerular filtration rate

### Nonlinear MR analysis

The distributions of the allele scores for eGFR values followed a normal distribution (Supplemental Fig. 1).

**Fig. 2:**
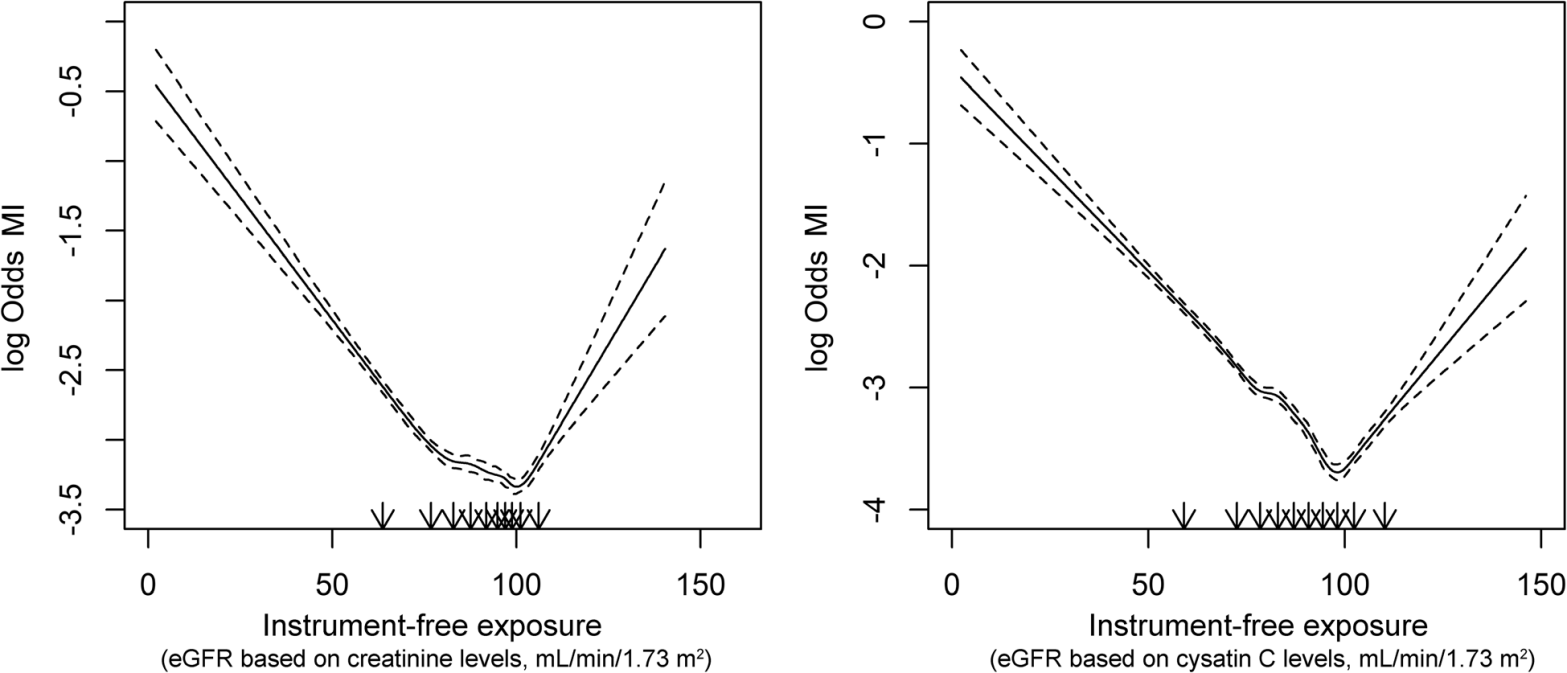
Restricted cubic spline curves. We used the instrument-free exposure as the exposure variable and the MI outcome as the outcome variable in logistic regression analysis. The cubic spline curves were plotted with 10 knots defined by deciles (black arrows). The left curve shows the results with eGFR values based on creatinine levels and the right curve shows the results with eGFR values based on cystatin C levels. The *y*-axes indicate the log odds ratios for MI

**Fig. 3:**
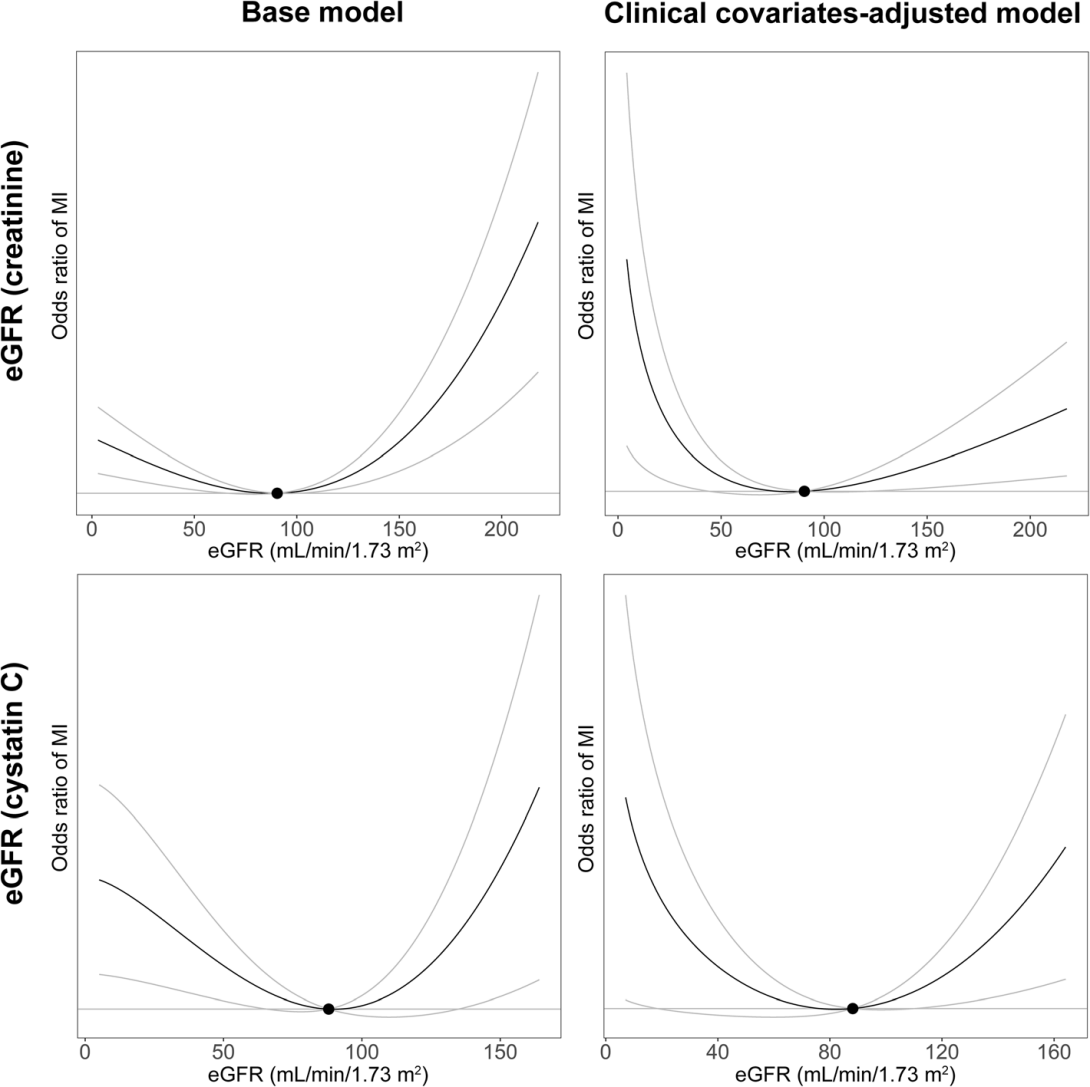
Results from the nonlinear Mendelian randomization investigation by fractional polynomial model. We used the fractional polynomial model of the degree 2 model with 100 strata. The base model included the adjusted covariates of age, sex, and the first 10 genetic principal components. The risk of MI according to creatinine-based eGFR or cystatin C-based eGFR, calculated by the CKD-EPI equation, was investigated in 321,024 individuals (12,205 MI cases). The clinical covariate-adjusted model was adjusted for body mass index, systolic blood pressure values, hypertension medication history, hemoglobin A1c level, history of diabetes diagnosis, levels of triglycerides, high-density lipoprotein, low-density lipoprotein, dyslipidemia medication history, and urine microalbumin. The sensitivity analysis was performed in 245,398 individuals (9128 MI cases) with complete information for the covariates. The black dots indicate the reference eGFR values (eGFR: 90.0 mL/min/1.73 m^2^)

We calculated localized averaged causal estimates by stratifying the population according to instrument-free exposure variables (Supplemental Table 3). The instrument-free variable showed U-shaped association with the risk of MI when we plotted cubic splines (Fig. 2). When genetically predicted creatinine-based eGFR was the exposure variable (Fig. 3 and Table 2), nonlinear MR analysis by fractional polynomial method demonstrated a quadratic, or a U-shaped, association (quadratic *P* value < 0.001) with MI risk, and the β1 (decreasing slope in low eGFR ranges) and β2 (increasing slope in high eGFR ranges) estimates were both significant. The results were similar even after clinical covariates were adjusted, and the slope was steeper in the low eGFR ranges where a higher genetically predicted eGFR was associated with a lower risk of MI.

**Table 2:** Meta-regression results of the causal estimates from nonlinear MR analysis by fractional polynomial method

Genetically predicted exposure	Adjusted covariates	Quadratic *P* value	β	Fractional polynomial model power	Estimated beta	Estimated standard error	Estimated *P* value
Creatinine-based eGFR	Age, sex, and 10 PCs	< 0.001	β1	1	− 5.36E−2	1.61E−3	< 0.001
			β2	3	2.31E−6	6.53E−7	< 0.001
	Age, sex, 10 PCs, clinical covariates (e.g., BMI, hypertension, diabetes, dyslipidemia, and albuminuria)	0.02	β1	0.5	− 8.87	3.57	0.013
			β2	log 0.5	1.38	0.55	0.013
Cystatin C-based eGFR	Age, sex, and 10 PCs	0.01	β1	2	− 1.48E−3	5.51E−4	0.007
			β2	log 2	2.96E−4	1.11E−4	0.008
	Age, sex, 10 PCs, clinical covariates (e.g., BMI, hypertension, diabetes, dyslipidemia, and albuminuria)	0.02	β1	0	− 1.44	0.67	0.03
			β2	3	8.85E−7	3.60E−7	0.01

Clinical covariates included in the adjusted model were body mass index, systolic blood pressure, hypertension medication history, diabetes mellitus diagnosis, hemoglobin A1c, medication history for dyslipidemia, triglycerides, high-density lipoprotein and low-density lipoprotein cholesterols, and urine microalbumin levels

*eGFR* estimated glomerular filtration rate, *PC* principal components, *BMI* body mass index

When the allele score for cystatin C-based eGFR was the exposure variable, a similar quadratic relation between genetically predicted eGFR and MI risk was identified, with both directions of causal estimates again being statistically significant. The results were similar when additional clinical covariates were adjusted for the model.

**Fig. 4:**
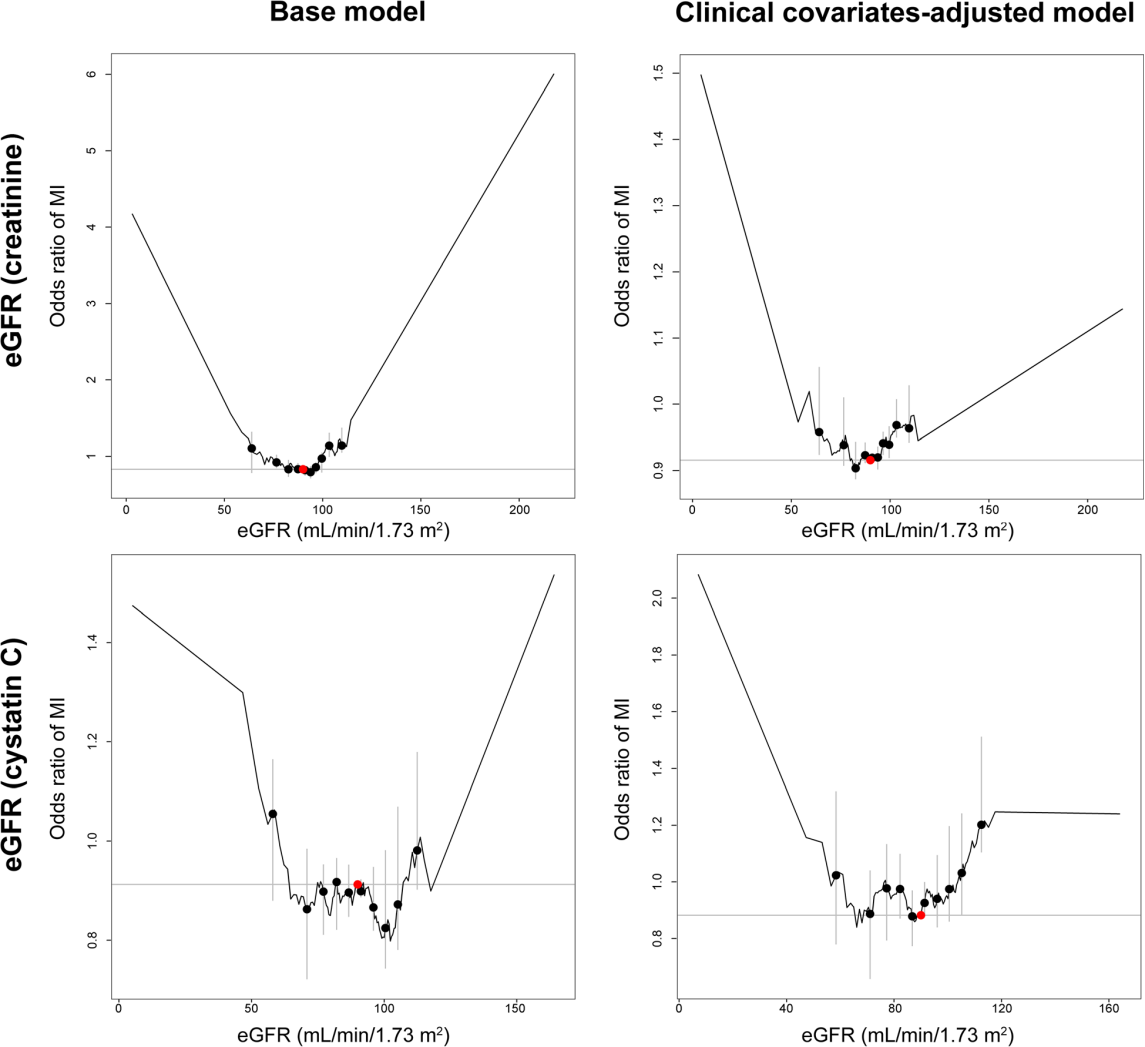
Results from the nonlinear Mendelian randomization investigation by piecewise linear method. We used the piecewise linear method with 100 strata. The base model included the adjusted covariates of age, sex, and the first 10 genetic principal components. The risk of MI according to creatinine-based eGFR or cystatin C-based eGFR, calculated by the CKD-EPI equation, was investigated in 321,024 individuals (12,205 MI cases). The clinical covariate-adjusted model was adjusted for body mass index, systolic blood pressure values, hypertension medication history, hemoglobin A1c level, history of diabetes diagnosis, levels of triglycerides, high-density lipoprotein, low-density lipoprotein, dyslipidemia medication history, and urine microalbumin. The sensitivity analysis was performed in 245,398 individuals (9128 MI cases) with complete information for the covariates. The red dots indicate the reference eGFR values (eGFR: 90.0 mL/min/1.73 m^2^)

The results by the piecewise linear method also demonstrated a U-shaped association for the causal estimates by eGFR on risks of MI (Fig. 4).

### Conventional summary-level MR analysis

When the conventional inverse variance weighted method under the linearity assumption was used to yield causal estimates by summary-level MR, the causal estimates remained null for both the creatinine- and cystatin C-based eGFR exposures on MI risk in the UK Biobank data (Table 3). Although no significant directional pleiotropy was suspected by MR-Egger intercept *P* values, the pleiotropy-robust summary-level MR sensitivity analyses also provided null causal estimates. The results were similar when the independent summary statistics from the CARDIoGRAMplusC4D consortium were used as the outcome data.

**Table 3:** Causal estimates from summary-level MR analysis under linearity assumption

Genetically predicted exposure	Outcome data	N of overlapping SNPs	MR-Egger intercept *P* value	MR methods	Estimated beta	Estimated standard error	Estimated *P* value
Creatinine-based eGFR	UK Biobank	140	0.359	Inverse variance weighted	− 0.121	0.544	0.824
				Weighted median	0.578	0.553	0.296
				MR-Egger	1.056	1.390	0.449
	CARDIoGRAMplusC4D	137	0.183	Inverse variance weighted	0.380	0.497	0.445
				Weighted median	0.279	0.510	0.585
				MR-Egger	1.985	1.297	0.128
Cystatin C-based eGFR	UK Biobank	347	0.618	Inverse variance weighted	− 0.289	0.350	0.409
				Weighted median	0.183	0.460	0.690
				MR-Egger	− 0.022	0.639	0.971
	CARDIoGRAMplusC4D	341	0.697	Inverse variance weighted	0.062	0.316	0.845
				Weighted median	− 0.286	0.376	0.448
				MR-Egger	− 0.135	0.598	0.821

*SNP* single-nucleotide polymorphism, *MR* Mendelian randomization, *eGFR* estimated glomerular filtration rate

## Discussion

In this MR study, we identified that genetically predicted eGFR is significantly associated with MI risk with a quadratic shape. Our results indicated that a reduction in eGFR may be a causal factor for higher MI risk in individuals with an eGFR in the low range. In addition, the results suggested that supranormal eGFR values, commonly known as kidney hyperfiltration, may causally elevate the risk of MI.

Kidney function impairment is one of the most widely recognized risk factors for cardiovascular diseases. Such a close linkage raised suspicion that kidney function impairment may causally increase the risk of MI. However, as there are shared risk factors such as hypertension and diabetes for CKD and MI and the possibility of reverse causation remains, the causal effects of kidney function on MI risk have been difficult to be confirmed by conventional observational studies. To solve this issue, recent studies have implemented MR analysis [8, 9]. In MR, causal estimates can be yielded as genetically predicted exposure is determined before birth; thus, the instrumental variable approach is minimally affected by confounding effects or reverse causation. However, previous MR results indicated null causal effects from cystatin C- or creatinine-related parameters and did not support that clinical interventions targeting kidney function impairment would also be helpful for reducing the risk of MI [8, 9]. However, previous MR analyses were based on the linearity assumption and tested the causal estimates throughout the entire range of kidney function exposure. As supranormal eGFR values were reported to be associated with all-cause mortality or atherosclerotic cardiovascular diseases, kidney function could have a parabolic causal effect on MI risk [4–6, 33, 34]. We implemented nonlinear MR analysis methods to investigate the issue and identified that genetically predicted eGFR was significantly associated with MI risk with a quadratic shape of the exposure-outcome relation. Therefore, this MR study supports that kidney function impairment would be a causal factor for a higher MI risk, and the linkage would not be from external confounding or reverse causal effects.

A reduction in eGFR, usually below 60 mL/min/1.73 m^2^ where stage 3 chronic kidney disease is defined [35], has been reported to be an independent risk factor for coronary artery disease. The observational associations remained significant even after adjustment for known traditional risk factors such as hypertension or diabetes [2]. Recent findings suggested the clinical significance of fibroblast-growth factor 23-mediated pathways or calcium-phosphate metabolism in regard to the risk of CKD progression and MI [36, 37]. A platelet-related mechanism has also been suggested to mediate the linkage between CKD and MI, as a reduction in eGFR was associated with higher thrombotic activity and poor responses to antiplatelet agents [38–40]. There have been other pathophysiologic mechanisms, such as the induction of inflammation, vascular calcification, or endothelial dysfunction, that may explain the close association between CKD and MI [41]. With the current MR findings, a decrease in eGFR below the reference range < 60 mL/min/1.73 m^2^, may be considered a “causal” factor that elevates the risk of MI. Further, the identified causal effects imply that clinical interventions targeting kidney function impairment may also be beneficial for preventing MI.

Kidney hyperfiltration has been reported to be associated with the risk of cardiovascular diseases [7, 11], even in a report where direct measurements of GFR were performed [42]. Specifically, a previous systematic meta-analysis including 24 observational cohorts of 637,315 individuals showed that eGFR ≥ 105 mL/min/1.73 m^2^ was significantly associated with higher risks of adverse cardiovascular risks [7]. Our results suggested that MI risk was higher in higher ranges of genetically predicted eGFR values, similar to previous observational findings, independent of major comorbidities, suggesting that kidney hyperfiltration may be another “causal” factor for MI similarly as the state of reduced eGFR below 60 mL/min/1.73 m^2^. This interpretation should be made carefully because eGFR is an estimated value and even cystatin C may be affected by nonkidney factors [43, 44]. However, as kidney hyperfiltration is considered another state of impaired kidney function associated with future rapid eGFR decline [45], it may be acceptable that early kidney function impairment represented as supranormal eGFR may affect MI risk. Upregulation of the renin-angiotensin-aldosterone system and increased proximal tubular sodium-glucose reabsorption are reasons for glomerular hyperfiltration as they affect tubuloglomerular feedback [46]. Considering that renin-angiotensin aldosterone system blockade or sodium-glucose cotransporter inhibitors 2 reduce both glomerular hyperfiltration and cardiovascular risk [47–50], the linkage may be explained by the mediating mechanism. A future study is warranted to validate our findings and confirm the mechanism of supranormal eGFR in regard to the risk of MI. In addition, a study may test the potential benefits of identifying the cause of kidney hyperfiltration or clinical interventions to reverse supranormal eGFR.

On the other hand, the parabolic shape of the association between genetically predicted eGFR and MI risk explained the null causal estimates reported by previous MR studies [8, 9] and our summary-level MR analysis. This study emphasizes that nonlinear MR analysis should be considered when a U-shaped causal estimate according to the exposure variable is suspected, as conventional summary-level MR analysis relies on the linearity assumption and can be attenuated for such quadrative relations. In addition, the overall effect size of the localized averaged causal estimates was relatively small compared to the findings in observational studies [7]. This finding may imply that the previously reported observational association between eGFR and MI risks might have been overestimated due to residual confounding effects.

There are some limitations of this study. First, MR analysis cannot prove the clinical utility of modifying an exposure to affect an outcome [51]. Although this study suggests the causal linkage between kidney function impairment and MI risk, a result based on a clinical trial is necessary to suggest the clinical implications of our findings. In addition, as it is difficult to provide interpretable effect sizes of the causal estimates in non-linear MR analysis, the degree of the suggested causal effects could not be determined herein. Second, MR analysis cannot provide a direct mechanistic explanation for the identified causal effects. In particular, as eGFR values are as an estimated value and cystatin C levels could also be affected by external factors, the mechanism of supranormal kidney hyperfiltration and its clinical significance should be validated in future studies. Third, the possibility of selection bias remains. Although the UK Biobank dataset is the largest genetic dataset where nonlinear MR analysis was possible, the UK Biobank cohort has a healthy volunteer bias [52]. Additional studies may be necessary to retest the causal estimates in a population with characteristics that are closer to those of the general population. Last, nonlinear MR studies rely on allele score-based analysis. Although we adjusted clinical covariates to support the attainment of the independence assumption, potential unmeasured pleiotropic effects should be considered.

## Conclusions

In conclusion, genetically predicted eGFR is significantly associated with the risk of MI with a parabolic shape, suggesting that kidney function impairment may causally elevate MI risk. Clinicians may pay attention to the measures to prevent kidney function impairment to reduce the risk of MI. Future study is warranted to investigate the clinical implications of the findings and the clinical significance of supranormal eGFR in regard to coronary artery disease risk.

## Supplementary Information

**Additional file 1:** Supplemental Methods. Detailed methods for the Mendelian randomization analysis. **Supplemental Figure 1**. Distribution of the phenotypical eGFR values and allele scores for log-transformed eGFR. **Supplemental Table 1**. Genetic instruments for log-transformed eGFR based on creatinine levels. **Supplemental Table 2**. Genetic instruments for log-transformed eGFR based on cystatin C levels. **Supplemental Table 3**. Localized averaged causal estimates calculated from 100 percentile ranges of strata according to the instrument-free exposure.

## Data Availability

The data for this study are available in the public domain, as described in the manuscript.

## Abbreviations

CKD: Chronic kidney disease
eGFR: Estimated glomerular filtration rate
MI: Myocardial infarction
MR: Mendelian randomization
SNP: Single nucleotide polymorphism
