# Supplementary material for: Nonlinear causal effects of estimated glomerular filtration rate on myocardial infarction risks: Mendelian randomization study
Source: BMC Med. 2022 Feb 3;20:44. doi: 10.1186/s12916-022-02251-1 (PMC8811984; doi:10.1186/s12916-022-02251-1)
Supplement: Supplementary file 1 — Additional file 1: Supplemental Methods. Detailed methods for the Mendelian randomization analysis. Supplemental Figure 1. Distribution of the phenotypical eGFR values and allele scores for log-transformed eGFR. Supplemental Table 1. Genetic instruments for log-transformed eGFR based on creatinine levels. Supplemental Table 2. Genetic instruments for log-transformed eGFR based on cystatin C levels. Supplemental Table 3. Localized averaged causal estimates calculated from 100 percentile ranges of strata according to the instrument-free exposure. [file 12916_2022_2251_MOESM1_ESM.pdf]

Nonlinear causal effects of estimated glomerular filtration rate on myocardial infarction risks: A Mendelian randomization study

Park et al.

Supplemental Material Table of Contents

Supplemental Methods.

Supplemental Figure 1. Distribution of the phenotypical eGFR values and allele scores for log-transformed eGFR.

Supplemental Table 1. Genetic instruments for log-transformed eGFR based on creatinine levels.

Supplemental Table 2. Genetic instruments for log-transformed eGFR based on cystatin C levels**.**

Supplemental Table 3. Localized averaged causal estimates calculated from 100 percentile ranges of strata according to the instrument-free exposure.

Supplemental Methods.

Data collection in the UK Biobank

The information of the data from the UK Biobank consortium are available online (URL: https://www.ukbiobank.ac.uk/data-showcase/), and the information is identified by field IDs.

Baseline eGFR values were calculated from the information of serum creatinine levels (Field ID 30700), cystatin C levels (Field ID 10720), or both, along with information of ethnicity (Field ID 21000), calculated by the CKD-EPI equation.

Other information was collected by as follows: age (Field ID 21003). sex (Field ID 31), body mass index (Field ID 21001), total cholesterol (Field ID 30690), HDL cholesterol (Field ID 30760), LDL cholesterol (Field ID 30780). Medication histories for hypertension or dyslipidemia (Field ID 6177 and 6153) were collected. Systolic and diastolic BP was determined by the average of two automated measurements (Field ID 4080 and Field ID 4079), and those with a single missing measurement were considered to have missing information. A history of diabetes mellitus was collected by self-reports (Field ID 2443). Glycated hemoglobin A1c levels (Field ID 30750) and urine albuminuria values (Field ID 30500) were also collected.

Methods for conventional summary-level MR analysis

The SNPs which overlapped with the outcome data were included in the conventional MR analysis under linearity assumption. Multiplicative random-effects inverse variance weighted method was used to yield the main causal estimates. MR-Egger regression was performed with calculation of MR-Egger intercept P value which is used to investigate the presence of significant directional pleiotropic effect. As MR-Egger regression can still be biased from violation of the InSIDE assumption, weighted-median method was implemented as another pleiotropy-robust sensitivity analysis. Weighted-median method has strength as the method can calculate valid causal estimates even half of the instrumented weights are invalid. The unit of the causal estimates were from an exponential increase in eGFR on log-odds for MI. All of the above commonly performed summary-level methods assume a linearity relation between exposure and outcome and cannot reflect non-linearity.

Supplemental Figure 1. Distribution of the phenotypical eGFR values and allele scores for log-transformed eGFR.

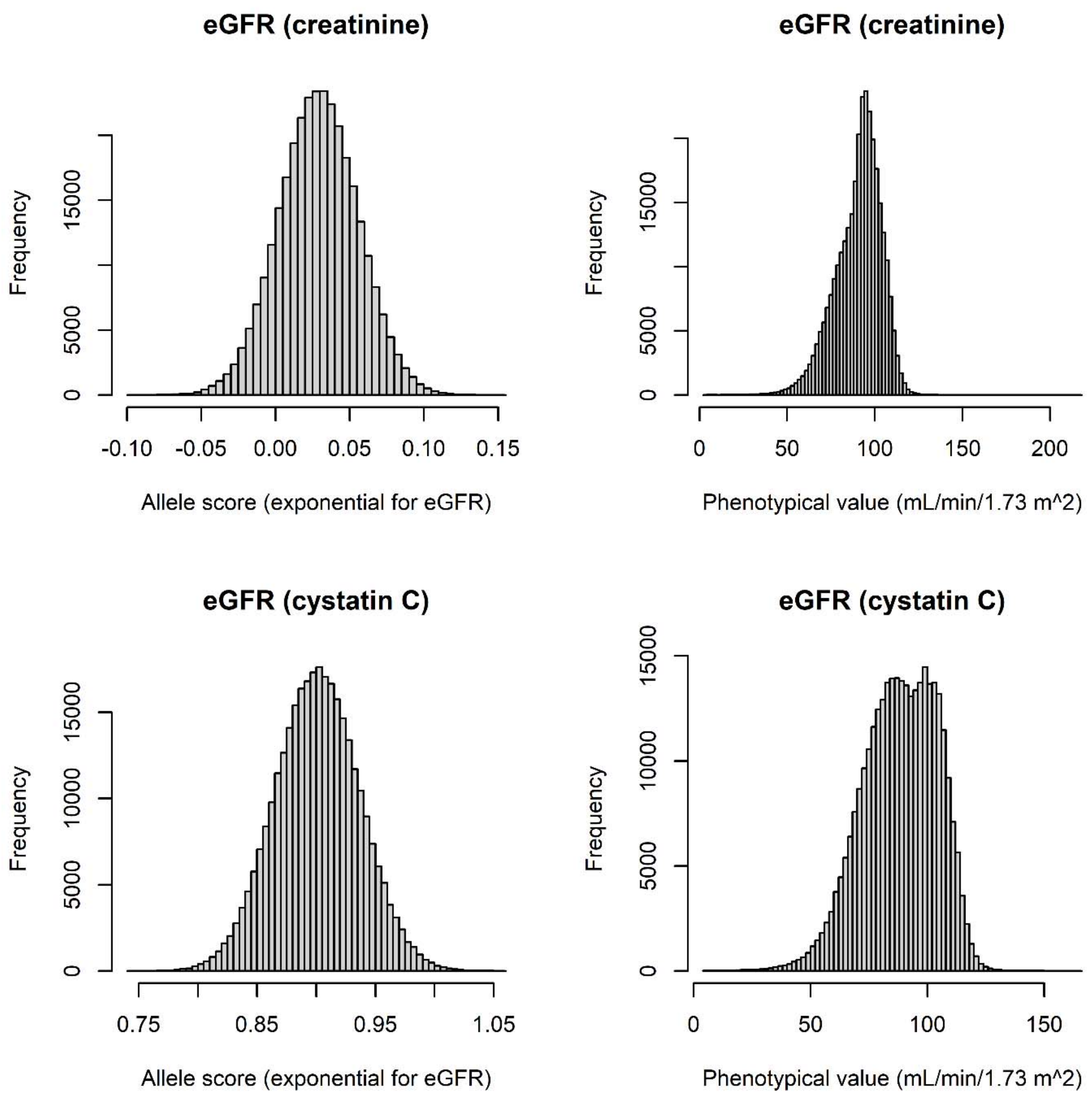

Supplemental Table 1. Genetic instruments for log-transformed eGFR based on creatinine levels.

Basic information			Variant filter	Exposure				eGFR (cystatin C)		
				eGFR (log-transformed, creatinine)						
RSID	effect allele	other allele	Irrelevant/weak association with eGFR (cystatin C)	beta	se	eaf	pval	beta	se	pval
rs1458038	T	C		0.0032	0.0004	0.3	3.60E-17	0.247	0.042	5.50E-09
rs2954017	T	C		0.0026	0.0004	0.46	3.20E-11	0.180	0.038	2.97E-06
rs10774625	A	G		-0.002	0.0003	0.48	1.80E-08	-0.996	0.038	1.11E-148
rs780094	T	C		0.0046	0.0004	0.38	4.20E-38	0.308	0.039	5.54E-15
rs77924615	A	G		0.0096	0.0005	0.2	1.20E-99	1.025	0.049	6.38E-99
rs154656	A	T		-0.0031	0.0003	0.44	1.30E-18	-0.262	0.039	1.70E-11
rs17696736	A	G		0.002	0.0004	0.57	9.70E-09	0.820	0.039	2.52E-99
rs7188071	T	C	TRUE	0.0024	0.0004	0.36	8.60E-12	-0.109	0.040	6.62E-03
rs10098664	T	C	TRUE	-0.0024	0.0004	0.51	6.00E-11	-0.070	0.039	7.00E-02
rs10838702	T	G		-0.0023	0.0004	0.38	6.30E-11	-0.223	0.039	1.65E-08
rs112545201	T	C		-0.0042	0.0005	0.13	8.80E-17	-0.242	0.056	1.55E-05
rs2411192	A	T		-0.0024	0.0003	0.59	5.80E-12	-0.234	0.039	2.24E-09
rs10096421	T	G	TRUE	0.002	0.0004	0.47	1.50E-08	0.125	0.039	1.20E-03
rs4625	A	G		-0.0023	0.0004	0.68	4.30E-10	-0.246	0.042	3.49E-09
rs223308	A	G		-0.0027	0.0003	0.52	3.00E-15	-0.371	0.038	4.18E-22
rs10224002	A	G		0.0068	0.0004	0.72	2.70E-66	0.851	0.042	3.37E-90
rs3134605	T	C		0.0033	0.0004	0.8	2.80E-13	0.444	0.046	1.03E-21
rs11784052	T	C	TRUE	0.0027	0.0004	0.46	9.80E-15	0.126	0.039	1.07E-03
rs11227260	T	G		-0.0032	0.0004	0.35	4.50E-19	-0.512	0.041	1.23E-36
rs7592697	T	C	TRUE	-0.002	0.0004	0.65	3.20E-08	-0.006	0.040	8.74E-01
rs72683923	T	C	TRUE	-0.0076	0.0014	0.98	2.00E-08	0.222	0.137	1.06E-01
rs7832708	T	C	TRUE	0.0023	0.0004	0.49	2.40E-10	0.092	0.038	1.64E-02
rs9828976	C	G		-0.0024	0.0004	0.75	2.00E-09	-0.349	0.045	9.32E-15
rs6029640	A	G	TRUE	-0.0021	0.0004	0.58	7.00E-09	-0.109	0.039	5.27E-03
rs35004449	T	G	TRUE	0.0027	0.0004	0.27	2.60E-12	0.093	0.044	3.41E-02
rs3905668	A	G		-0.0025	0.0004	0.72	2.80E-11	-0.398	0.043	1.87E-20
rs281380	T	C		-0.0022	0.0004	0.63	1.90E-09	-0.157	0.040	8.74E-05
rs144100226	T	C	TRUE	0.006	0.0011	0.04	1.30E-08	0.282	0.091	1.96E-03
rs1910738	T	G	TRUE	0.0023	0.0004	0.71	1.20E-08	0.026	0.043	5.49E-01
rs12207180	A	T	TRUE	-0.0085	0.0005	0.12	1.20E-56	-0.080	0.057	1.59E-01
rs78614739	T	C	TRUE	0.0026	0.0005	0.17	2.10E-08	-0.104	0.052	4.56E-02
rs2823139	A	G		-0.0027	0.0004	0.34	1.00E-13	-0.387	0.041	1.95E-21
rs7012814	A	G	TRUE	0.0025	0.0004	0.47	1.80E-12	0.064	0.039	9.94E-02
rs1543238	A	G	TRUE	-0.0025	0.0004	0.53	3.20E-12	-0.125	0.039	1.25E-03
rs11657044	T	C		-0.0075	0.0005	0.17	5.30E-60	-0.310	0.052	2.64E-09
rs1051447	A	C	TRUE	0.0021	0.0004	0.71	2.60E-08	0.021	0.042	6.21E-01
rs2068888	A	G		-0.0026	0.0003	0.45	6.30E-14	-0.327	0.039	1.92E-17
rs881858	A	G		-0.0056	0.0004	0.7	1.10E-49	-0.628	0.041	1.15E-51
rs419291	T	C	TRUE	0.0021	0.0004	0.39	4.10E-09	0.106	0.040	8.21E-03
rs12920176	A	C		-0.0026	0.0004	0.59	2.40E-13	-0.278	0.039	1.13E-12
rs6921580	C	G		0.0027	0.0004	0.41	1.60E-14	0.238	0.039	1.01E-09
rs56140069	A	T		0.0025	0.0005	0.82	2.70E-08	0.361	0.050	3.01E-13
rs6492982	T	C		-0.0032	0.0004	0.55	5.80E-19	-0.251	0.039	9.61E-11
rs3111316	A	G	TRUE	-0.0019	0.0004	0.59	4.90E-08	0.036	0.039	3.55E-01
rs72817412	T	C		0.0049	0.0009	0.05	1.50E-08	0.792	0.090	1.79E-18
rs2792796	T	C		-0.0021	0.0004	0.61	3.10E-09	-0.195	0.039	6.75E-07
rs55759218	A	G		-0.0039	0.0004	0.27	6.10E-24	-0.466	0.043	1.10E-27
rs2267372	A	G		0.0024	0.0004	0.4	1.20E-11	0.220	0.039	2.01E-08
rs1047891	A	C	TRUE	-0.0065	0.0004	0.31	3.60E-64	0.430	0.041	2.13E-25

rs4808154	T	C	TRUE	0.0026	0.0004	0.71	7.80E-09	0.005	0.042	9.11E-01
rs4410790	T	C	TRUE	-0.0023	0.0004	0.37	1.90E-10	-0.148	0.040	2.01E-04
rs7974833	T	C		-0.0032	0.0004	0.76	4.00E-15	-0.452	0.045	1.28E-23
rs17413465	A	C	TRUE	0.0025	0.0004	0.18	1.40E-08	0.101	0.049	3.98E-02
rs6971211	T	C		-0.0029	0.0004	0.41	3.10E-15	-0.330	0.042	2.85E-15
rs2472297	T	C		0.0039	0.0004	0.26	8.20E-20	0.313	0.043	4.92E-13
rs1268176	A	G		0.0027	0.0004	0.34	7.10E-14	0.173	0.041	2.64E-05
rs3925584	T	C		-0.0055	0.0003	0.55	3.00E-56	-0.596	0.038	3.49E-54
rs1028455	A	T	TRUE	0.0021	0.0004	0.33	1.90E-08	0.110	0.041	7.33E-03
rs688540	A	G		-0.0031	0.0006	0.87	2.30E-08	-0.305	0.056	4.35E-08
rs6484504	T	C		-0.0032	0.0004	0.28	1.20E-16	-0.248	0.043	7.59E-09
rs363092	A	C	TRUE	-0.0022	0.0004	0.42	5.60E-10	0.031	0.039	4.28E-01
rs1377164	T	C	TRUE	0.0034	0.0004	0.21	9.80E-16	-0.046	0.047	3.30E-01
rs2490391	A	C		-0.0025	0.0003	0.46	5.50E-13	-0.250	0.039	9.49E-11
rs3845534	A	G		-0.0019	0.0003	0.49	3.90E-08	-0.248	0.038	1.15E-10
rs9907229	T	C		-0.0049	0.0005	0.85	6.80E-24	-0.646	0.054	1.97E-32
rs736820	A	G		-0.0021	0.0004	0.37	5.30E-09	-0.154	0.040	1.04E-04
rs13230509	C	G		-0.0055	0.0004	0.69	4.00E-37	-0.642	0.043	8.48E-51
rs6722113	A	G	TRUE	-0.0022	0.0004	0.34	1.30E-08	-0.150	0.041	2.72E-04
rs1635404	T	G	TRUE	-0.0024	0.0004	0.7	2.50E-10	-0.148	0.042	3.68E-04
rs325442	A	G		0.0021	0.0003	0.4	3.00E-09	0.272	0.039	2.89E-12
rs17462630	C	G		0.0024	0.0004	0.34	4.30E-09	0.395	0.041	2.06E-22
rs112880707	T	C	TRUE	0.0056	0.0006	0.11	6.70E-23	0.042	0.066	5.30E-01
rs2337143	A	G		-0.0021	0.0004	0.34	5.60E-09	-0.173	0.041	2.34E-05
rs11564722	T	C		0.0038	0.0004	0.24	1.40E-18	0.458	0.046	1.07E-23
rs1913641	T	G	TRUE	-0.002	0.0003	0.48	4.80E-09	-0.077	0.038	4.35E-02
rs7169629	C	G	TRUE	0.0019	0.0003	0.52	2.40E-08	0.083	0.038	3.01E-02
rs9838792	A	G	TRUE	0.0031	0.0004	0.39	4.80E-19	0.006	0.039	8.77E-01
rs62257807	T	C		-0.0046	0.0008	0.06	2.90E-08	-0.400	0.077	2.22E-07
rs2634675	A	G	TRUE	0.0028	0.0004	0.46	5.30E-13	0.029	0.039	4.47E-01
rs10964603	T	C	TRUE	-0.0025	0.0004	0.78	7.80E-09	-0.160	0.047	6.72E-04
rs79760705	T	G		0.0056	0.0006	0.11	2.60E-24	0.465	0.061	1.74E-14
rs632887	A	G	TRUE	0.0033	0.0004	0.59	1.10E-20	0.008	0.039	8.37E-01
rs407102	T	C	TRUE	0.0031	0.0004	0.7	3.80E-16	0.082	0.042	5.21E-02
rs9375694	A	G		0.0026	0.0004	0.7	4.60E-12	0.191	0.042	4.77E-06
rs9807656	T	C	TRUE	-0.0034	0.0006	0.9	3.80E-09	0.006	0.064	9.26E-01
rs7127946	T	C	TRUE	0.0023	0.0004	0.72	1.80E-09	0.132	0.042	1.74E-03
rs1887252	C	G		-0.0029	0.0004	0.64	7.40E-16	-0.314	0.040	4.29E-15
rs62257555	A	G		0.0048	0.0009	0.94	2.60E-08	0.392	0.080	8.81E-07
rs80576	A	G	TRUE	-0.0027	0.0005	0.16	1.00E-08	-0.187	0.051	2.65E-04
rs4886699	A	C		0.0031	0.0004	0.75	4.00E-15	0.309	0.044	2.47E-12
rs168505	T	C		-0.0027	0.0004	0.4	2.20E-14	-0.278	0.039	1.54E-12
rs499600	T	G		-0.0037	0.0005	0.15	8.90E-15	-0.444	0.052	2.56E-17
rs12163971	A	C	TRUE	-0.0032	0.0005	0.16	4.30E-12	0.010	0.052	8.49E-01
rs2509851	A	C	TRUE	0.0021	0.0004	0.63	1.60E-09	0.117	0.040	3.17E-03
rs66473811	T	C	TRUE	0.0031	0.0005	0.84	2.00E-10	0.128	0.052	1.30E-02
rs6667182	T	C		-0.0043	0.0004	0.32	2.20E-23	-0.288	0.041	3.58E-12
rs76215063	T	C	TRUE	-0.0041	0.0007	0.92	2.60E-09	-0.117	0.079	1.36E-01
rs6779368	A	G	TRUE	0.0033	0.0004	0.66	8.80E-16	0.002	0.040	9.51E-01
rs8096658	C	G		0.0046	0.0004	0.51	1.80E-29	0.419	0.039	3.70E-27
rs11951093	A	G		-0.0056	0.0004	0.42	2.00E-54	-0.349	0.039	5.59E-19
rs6948759	T	C	TRUE	-0.0026	0.0004	0.21	1.00E-09	-0.177	0.048	2.48E-04
rs7543734	C	G		0.0031	0.0005	0.2	9.60E-11	0.201	0.048	2.31E-05
rs10430743	T	G		0.0025	0.0003	0.43	2.90E-13	0.461	0.039	1.19E-32
rs72841902	A	T	TRUE	0.0022	0.0004	0.29	3.60E-09	0.016	0.043	7.07E-01
rs10122824	T	G	TRUE	-0.0024	0.0004	0.34	2.80E-10	0.244	0.041	2.21E-09
rs7084764	A	G	TRUE	0.0026	0.0003	0.5	2.90E-14	0.141	0.038	2.46E-04

rs7651407	T	C		0.0027	0.0004	0.45	1.60E-11	0.236	0.038	8.79E-10
rs3757387	T	C		0.0029	0.0004	0.55	2.50E-16	0.635	0.039	5.71E-61
rs75267082	A	T	TRUE	0.0034	0.0006	0.89	1.10E-09	0.144	0.061	1.78E-02
rs2261092	A	G	TRUE	-0.0045	0.0007	0.07	1.40E-09	-0.243	0.070	5.20E-04
rs12361687	A	G		0.0021	0.0004	0.36	5.00E-09	0.150	0.040	1.44E-04
rs187355703	C	G		0.0101	0.0011	0.97	9.50E-19	1.261	0.122	6.70E-25
rs6546869	A	G		0.0061	0.0004	0.22	1.70E-48	0.455	0.046	6.80E-23
rs4656220	T	C	TRUE	0.0021	0.0004	0.37	1.60E-09	-0.004	0.040	9.30E-01
rs2365286	A	G		-0.0033	0.0004	0.74	2.10E-17	-0.360	0.044	2.76E-16
rs3791221	A	G		0.0021	0.0004	0.65	3.00E-09	0.411	0.040	1.48E-24
rs303937	A	T		0.0027	0.0004	0.41	2.90E-14	0.438	0.039	7.77E-29
rs10851885	A	G		0.005	0.0004	0.76	3.30E-34	0.672	0.044	7.43E-52
rs28817415	T	C		-0.0074	0.0003	0.44	9.70E-104	-1.118	0.038	3.25E-186
rs9375818	A	G	TRUE	-0.0026	0.0004	0.23	3.20E-10	-0.093	0.046	4.44E-02
rs6501468	T	C	TRUE	0.0024	0.0004	0.23	1.40E-08	-0.026	0.046	5.75E-01
rs3812036	T	C		-0.0069	0.0004	0.26	3.20E-64	-0.782	0.045	1.08E-67
rs2235826	A	T	TRUE	-0.0033	0.0005	0.81	3.90E-13	-0.178	0.050	3.30E-04
rs2071047	A	G	TRUE	0.002	0.0003	0.41	1.10E-08	0.143	0.039	2.60E-04
rs7966357	C	G		0.0024	0.0004	0.66	6.00E-11	0.165	0.040	3.58E-05
rs6127099	A	T		-0.0051	0.0004	0.72	1.20E-36	-0.561	0.044	1.26E-37
rs10865189	C	G		0.0025	0.0004	0.47	1.00E-12	0.237	0.039	8.53E-10
rs9868185	A	G		0.0027	0.0003	0.54	1.50E-14	0.318	0.038	1.25E-16
rs816828	T	C	TRUE	-0.002	0.0004	0.51	2.40E-08	0.012	0.038	7.53E-01
rs988911	A	G	TRUE	-0.0029	0.0005	0.13	1.40E-08	0.037	0.056	5.17E-01
rs11919484	T	G		-0.0023	0.0004	0.31	4.00E-10	-0.350	0.042	3.86E-17
rs11260709	T	C	TRUE	0.0024	0.0004	0.68	1.90E-10	0.132	0.041	1.18E-03
rs4794814	A	G	TRUE	-0.0059	0.0004	0.75	5.00E-49	-0.117	0.044	7.98E-03
rs11063193	T	C		0.0029	0.0005	0.88	4.90E-08	0.241	0.058	3.75E-05
rs1242484	T	C	TRUE	-0.0025	0.0004	0.69	8.80E-11	-0.007	0.042	8.58E-01
rs41284816	T	G		-0.0079	0.0012	0.03	1.60E-10	-0.643	0.142	6.16E-06
rs12520984	C	G	TRUE	0.0022	0.0004	0.33	2.40E-09	0.139	0.041	7.68E-04
rs71606723	A	T	TRUE	0.0029	0.0004	0.76	9.30E-13	0.087	0.046	6.02E-02
rs80282103	A	T		0.0081	0.0006	0.92	2.60E-37	0.838	0.070	2.03E-33
rs284859	T	G	TRUE	0.0027	0.0004	0.19	1.50E-09	0.176	0.050	4.68E-04
rs7185391	T	G	TRUE	-0.0026	0.0004	0.29	1.20E-11	-0.024	0.043	5.83E-01
rs700753	C	G		0.0033	0.0004	0.34	7.50E-20	0.454	0.040	1.88E-29
rs956006	T	C	TRUE	0.0022	0.0004	0.34	5.70E-09	0.113	0.041	5.76E-03
rs12913015	T	C		0.0028	0.0004	0.44	4.60E-15	0.295	0.039	2.83E-14
rs12989250	A	G		-0.0026	0.0004	0.31	1.20E-11	-0.299	0.042	9.02E-13
rs11694902	A	G		0.0041	0.0005	0.14	2.10E-16	0.452	0.055	3.17E-16
rs7838146	T	C		-0.0021	0.0004	0.36	1.20E-08	-0.273	0.040	9.26E-12
rs7514450	T	C		0.0022	0.0003	0.43	1.50E-10	0.150	0.039	1.16E-04
rs1783827	A	G	TRUE	-0.0021	0.0004	0.57	5.90E-09	-0.106	0.039	6.09E-03
rs140179699	A	G		0.0073	0.0011	0.95	1.20E-11	1.103	0.100	4.52E-28
rs1570521	T	G		0.002	0.0004	0.41	8.50E-09	0.164	0.039	2.53E-05
rs4441471	A	G	TRUE	0.0022	0.0004	0.72	1.10E-08	0.127	0.042	2.78E-03
rs6555317	A	G		0.0024	0.0004	0.69	6.40E-09	0.248	0.041	1.93E-09
rs7095954	A	T		-0.0019	0.0003	0.47	2.80E-08	-0.216	0.038	1.71E-08
rs35662455	C	G	TRUE	0.003	0.0005	0.88	2.90E-08	-0.020	0.060	7.36E-01
rs6088734	A	T	TRUE	0.0029	0.0003	0.43	6.60E-17	0.002	0.039	9.49E-01
rs11211257	A	G		0.0039	0.0006	0.9	2.10E-11	0.448	0.062	3.55E-13
rs6135224	A	G	TRUE	-0.002	0.0004	0.69	4.00E-08	-0.080	0.042	5.29E-02
rs1050816	T	C	TRUE	0.0029	0.0004	0.33	1.70E-15	0.083	0.041	4.23E-02
rs11071738	T	C		-0.0025	0.0003	0.53	5.50E-13	-0.335	0.039	3.74E-18
rs11794652	A	G	TRUE	-0.0028	0.0005	0.16	9.30E-09	-0.164	0.052	1.64E-03
rs9894634	T	C		-0.0021	0.0003	0.6	1.70E-09	-0.164	0.039	2.80E-05
rs2244237	T	G		0.0027	0.0004	0.22	8.40E-11	0.302	0.046	4.18E-11

rs62432759	A	G		-0.0025	0.0004	0.78	7.60E-09	-0.176	0.047	1.92E-04
rs7535253	T	C		0.0023	0.0004	0.21	4.90E-08	0.191	0.047	4.84E-05
rs1119066	A	C		0.0027	0.0005	0.15	2.00E-08	0.230	0.053	1.60E-05
rs4567937	A	G		-0.0032	0.0004	0.32	6.40E-18	-0.425	0.041	6.54E-25
rs111827672	A	T		0.0031	0.0004	0.32	1.00E-16	0.259	0.040	1.64E-10
rs1153855	C	G	TRUE	0.0086	0.0004	0.62	1.20E-132	0.006	0.040	8.87E-01
rs7203398	A	C		0.0027	0.0004	0.73	2.90E-12	0.381	0.043	5.85E-19
rs78444298	A	G		-0.0107	0.0014	0.02	2.50E-14	-0.735	0.139	1.32E-07
rs11071939	T	C	TRUE	-0.0038	0.0007	0.92	4.50E-09	-0.040	0.075	5.98E-01
rs3850625	A	G	TRUE	0.0048	0.0006	0.12	3.60E-18	0.163	0.059	5.91E-03
rs7565830	A	G		-0.0022	0.0004	0.72	8.80E-09	-0.210	0.043	1.08E-06
rs13029395	T	C		0.0034	0.0006	0.18	1.50E-09	0.199	0.051	8.54E-05
rs78936994	T	G		0.0024	0.0004	0.22	1.00E-08	0.179	0.047	1.37E-04
rs55938024	A	G		-0.0065	0.0006	0.12	1.40E-26	-0.582	0.062	4.34E-21
rs4566	T	G	TRUE	0.002	0.0004	0.61	1.00E-08	0.067	0.039	8.68E-02
rs7326821	A	G		0.0026	0.0005	0.83	3.80E-08	0.436	0.050	5.56E-18
rs10086569	T	C		0.0028	0.0004	0.24	5.70E-12	0.184	0.046	5.37E-05
rs495237	T	G		0.0029	0.0004	0.25	5.50E-13	0.166	0.044	1.45E-04
rs506000	T	C		-0.0038	0.0006	0.91	4.20E-10	-0.458	0.070	4.62E-11
rs35472707	T	C		-0.0075	0.0008	0.05	9.50E-20	-0.461	0.090	2.96E-07
rs11261022	A	C	TRUE	-0.0027	0.0004	0.36	3.70E-14	-0.128	0.040	1.43E-03
rs1595810	A	G		-0.0024	0.0004	0.2	4.40E-08	-0.255	0.048	1.31E-07
rs1858800	T	C		0.0022	0.0004	0.35	3.70E-09	0.156	0.041	1.18E-04
rs34468415	A	G	TRUE	-0.0028	0.0004	0.64	2.80E-15	-0.109	0.040	6.02E-03
rs3822939	A	G	TRUE	-0.0028	0.0003	0.46	3.10E-16	0.046	0.038	2.36E-01
rs6458868	T	C		-0.0021	0.0004	0.65	3.50E-09	-0.278	0.040	4.10E-12
rs12736457	C	G	TRUE	0.0056	0.0005	0.87	8.80E-27	0.064	0.057	2.68E-01
rs3793805	A	G	TRUE	-0.002	0.0004	0.57	1.00E-08	-0.123	0.039	1.50E-03
rs35629566	C	G	TRUE	0.003	0.0005	0.83	5.70E-10	-0.140	0.050	5.49E-03
rs2074204	T	C	TRUE	-0.0025	0.0004	0.26	2.20E-10	-0.056	0.044	2.04E-01
rs4886425	A	G	TRUE	-0.0027	0.0005	0.17	4.30E-09	-0.099	0.051	5.30E-02
rs509345	A	G		0.0024	0.0003	0.52	1.30E-12	0.285	0.038	1.11E-13
rs417237	T	G	TRUE	0.002	0.0004	0.61	1.10E-08	0.019	0.040	6.35E-01
rs11062167	A	G	TRUE	-0.0042	0.0003	0.53	7.10E-34	-0.099	0.039	1.01E-02
rs13157326	A	G		-0.0027	0.0004	0.48	2.90E-12	-0.220	0.039	1.62E-08
rs6088528	A	G	TRUE	-0.0033	0.0003	0.5	9.60E-22	-0.091	0.038	1.76E-02
rs544169	A	G	TRUE	0.0024	0.0004	0.74	1.00E-09	-0.041	0.043	3.41E-01
rs35072105	A	G	TRUE	-0.0021	0.0004	0.55	2.00E-09	-0.102	0.038	7.84E-03
rs1397764	A	G		0.0047	0.0004	0.28	7.80E-34	0.270	0.043	4.52E-10
rs396341	T	C	TRUE	0.003	0.0004	0.26	1.70E-14	0.161	0.044	2.37E-04
rs9887775	A	G	TRUE	-0.0035	0.0004	0.81	5.20E-15	-0.158	0.050	1.51E-03
rs11237450	A	C	TRUE	0.003	0.0005	0.17	2.10E-09	-0.137	0.052	8.78E-03
rs10994860	T	C		0.0039	0.0004	0.19	2.70E-18	0.396	0.050	4.14E-15
rs72995641	A	G		-0.0026	0.0004	0.2	1.40E-09	-0.208	0.048	1.26E-05
rs2156664	T	C	TRUE	-0.0021	0.0004	0.27	3.80E-08	-0.149	0.043	5.71E-04
rs267738	T	G		-0.005	0.0004	0.79	1.30E-32	-0.523	0.046	1.20E-29
rs3797537	A	G	TRUE	0.0021	0.0004	0.71	1.90E-08	0.140	0.043	1.00E-03
rs7667050	T	C	TRUE	0.002	0.0003	0.47	3.00E-09	0.108	0.038	4.90E-03
rs11166440	A	G		0.0021	0.0004	0.63	1.10E-08	0.270	0.040	1.30E-11
rs2442604	T	C	TRUE	-0.002	0.0003	0.55	9.20E-09	-0.055	0.039	1.52E-01
rs72834794	A	C	TRUE	-0.0041	0.0006	0.91	2.00E-10	-0.181	0.070	1.03E-02
rs62491533	T	C		-0.0027	0.0005	0.83	2.10E-09	-0.201	0.051	8.41E-05
rs61993680	A	C	TRUE	-0.0022	0.0004	0.65	1.50E-08	-0.122	0.040	2.36E-03
rs10851543	A	G		0.003	0.0003	0.56	1.30E-18	0.413	0.039	1.18E-26
rs2301343	T	G	TRUE	-0.0023	0.0004	0.74	9.10E-09	-0.105	0.045	1.97E-02
rs4836732	T	C		0.0025	0.0003	0.53	4.70E-13	0.215	0.039	2.54E-08
rs1719934	A	G	TRUE	0.0028	0.0003	0.54	3.70E-16	0.114	0.039	3.06E-03

rs2252281	T	C	TRUE	0.0041	0.0004	0.61	8.50E-30	0.096	0.039	1.52E-02
rs12024377	A	G		0.002	0.0004	0.37	4.20E-08	0.192	0.040	1.59E-06
rs113956264	T	C	TRUE	0.0081	0.0012	0.04	1.70E-11	0.005	0.112	9.64E-01
rs117113238	A	G		0.0039	0.0006	0.09	1.10E-10	0.555	0.068	5.57E-16
rs11191686	A	G	TRUE	0.002	0.0004	0.36	3.70E-08	0.065	0.040	1.04E-01
rs1509117	A	T	TRUE	0.0025	0.0004	0.3	3.30E-10	0.067	0.043	1.20E-01
rs1883991	A	C	TRUE	-0.0032	0.0004	0.69	2.30E-17	-0.132	0.041	1.23E-03
rs3795503	T	C		0.0022	0.0004	0.33	1.50E-08	0.223	0.041	6.43E-08
rs10272546	A	G	TRUE	-0.002	0.0003	0.54	7.90E-09	-0.092	0.038	1.66E-02
rs77915916	A	T	TRUE	0.0047	0.0006	0.92	3.50E-14	-0.109	0.069	1.14E-01
rs1548945	T	C	TRUE	0.0037	0.0004	0.41	1.30E-24	0.095	0.039	1.53E-02
rs10846157	A	C		-0.0036	0.0004	0.81	1.30E-16	-0.480	0.048	1.06E-23
rs113445505	T	C		0.0038	0.0004	0.37	2.00E-26	0.342	0.039	3.40E-18
rs10432479	T	C	TRUE	-0.0021	0.0004	0.37	4.20E-09	-0.093	0.040	1.94E-02
rs807624	T	G		0.0034	0.0004	0.34	1.50E-20	0.549	0.040	6.18E-43
rs233438	A	G		0.0043	0.0004	0.81	2.80E-22	0.227	0.050	4.79E-06
rs13200335	A	C		0.0024	0.0003	0.42	1.50E-11	0.196	0.039	5.11E-07
rs62187541	A	G		-0.0037	0.0007	0.93	4.10E-08	-0.323	0.078	3.12E-05
rs62053077	T	G	TRUE	-0.0025	0.0004	0.37	3.90E-10	0.112	0.040	4.66E-03
rs4871905	C	G		-0.0043	0.0003	0.42	1.80E-35	-0.708	0.039	7.91E-74
rs6833292	T	C	TRUE	0.002	0.0003	0.44	1.10E-08	0.093	0.039	1.60E-02
rs8101667	T	C		0.005	0.0004	0.33	2.20E-43	0.509	0.041	4.14E-36
rs2039424	A	G		0.0048	0.0004	0.62	9.70E-41	0.400	0.040	5.47E-24
rs78986840	T	C	TRUE	-0.0045	0.0007	0.94	1.40E-09	-0.064	0.081	4.30E-01
rs3018667	A	G		-0.0024	0.0004	0.32	1.20E-10	-0.178	0.041	1.08E-05
rs2834317	A	G		-0.0031	0.0005	0.15	2.80E-10	-0.540	0.054	6.02E-24
rs73116829	A	G		-0.0043	0.0006	0.11	1.60E-13	-0.490	0.067	3.16E-13
rs6481598	C	G		0.0023	0.0004	0.78	3.80E-08	0.269	0.047	1.12E-08
rs7687209	T	C	TRUE	0.0022	0.0004	0.42	1.50E-09	0.085	0.039	2.79E-02
rs6780429	A	C	TRUE	-0.002	0.0003	0.53	6.70E-09	0.052	0.039	1.74E-01
rs1994887	A	C		-0.0024	0.0004	0.28	2.30E-09	-0.201	0.044	4.09E-06
rs1569011	A	G	TRUE	0.002	0.0003	0.44	1.70E-08	0.044	0.039	2.58E-01
rs57126710	T	C	TRUE	0.0025	0.0004	0.35	6.20E-12	0.148	0.040	2.10E-04
rs28581385	A	T		-0.0033	0.0005	0.85	1.60E-11	-0.397	0.055	3.88E-13
rs2236521	A	G		-0.0022	0.0004	0.55	4.80E-10	-0.156	0.039	5.40E-05
rs28404308	A	T		0.0027	0.0005	0.62	2.00E-09	NA	NA	NA

eGFR = estimated glomerular filtration rate, se = standard error, pval = P value, OR = odds ratio

Supplemental Table 2. Genetic instruments for log-transformed eGFR based on cystatin C levels.

Basic information			Variant filter	Exposure					log-transformed eGFR (creatinine)					Blood urea nitrogen				
				log-transformed eGFR (cystatin C)														
RSID	effect allele	other allele	Irrelevant, weak association with alternate kidney function marker)	eaf	beta	se	pval	sample size	eaf	beta	se	pval	sample size	eaf	beta	se	pval	sample size
rs10015716	G	A		0.6448	-0.0015	5.00E-04	0.00127	460826	0.3578	-0.0015	3.00E-04	7.01E-09	1158670	0.6429	7.00E-04	5.00E-04	0.19	809561
rs10035662	C	A		0.6713	-0.0012	5.00E-04	0.01474	460826	0.3196	-0.0015	3.00E-04	3.05E-09	1145780	0.6834	7.00E-04	5.00E-04	0.196	835094
rs10040082	C	T		0.378	-0.0013	5.00E-04	0.004849	460826	0.6104	-0.0018	3.00E-04	8.87E-12	1102830	0.3993	0.0026	5.00E-04	1.37E-07	805066
rs1004441	G	A		0.7478	-0.0019	5.00E-04	0.000438	460826	0.2509	-0.0018	3.00E-04	3.07E-11	1201730	0.7528	-4.00E-04	6.00E-04	0.4943	852479
rs10111039	G	A	TRUE	0.5855	-8.00E-04	5.00E-04	0.08943	460826	0.4263	-0.0015	3.00E-04	1.33E-08	1196300	0.5608	-7.00E-04	5.00E-04	0.1444	851729
rs10142359	G	A		0.5079	-0.002	5.00E-04	1.52E-05	460826	0.5083	-0.0021	3.00E-04	1.85E-16	1201880	0.4905	1.00E-04	5.00E-04	0.8277	852635
rs10151563	A	G		0.6137	-0.0022	5.00E-04	2.02E-06	460826	0.6208	-0.0018	3.00E-04	1.09E-12	1201700	0.624	0.0014	5.00E-04	0.005893	852452
rs10182296	G	A	TRUE	0.6288	-7.00E-04	5.00E-04	0.1192	460826	0.3599	-0.002	3.00E-04	9.22E-15	1201830	0.6414	1.00E-04	5.00E-04	0.9092	852581
rs10206899	T	C		0.7777	-0.0051	6.00E-04	2.62E-20	460826	0.7745	-0.0063	3.00E-04	3.32E-84	1040520	0.7727	0.0034	6.00E-04	7.00E-08	704884
rs10224210	C	T		0.7224	-0.0092	5.00E-04	2.04E-72	460826	0.2773	-0.0078	3.00E-04	1.57E-139	978562	0.7202	0.0127	6.00E-04	9.13E-97	648247
rs10241879	T	A		0.6053	-0.0026	5.00E-04	3.75E-08	460826	0.3829	-0.0017	3.00E-04	2.61E-11	1201780	0.6218	0.0015	5.00E-04	0.002283	852532
rs10283362	C	T		0.1588	-0.0027	6.00E-04	1.85E-05	460826	0.8416	-0.0021	3.00E-04	4.94E-10	1159120	0.1576	0.0025	7.00E-04	0.000327	810011
rs1041606	T	C		0.2473	-0.0013	5.00E-04	0.01787	460826	0.2407	-0.0018	3.00E-04	8.70E-09	1198730	0.2394	0.002	6.00E-04	0.000807	850688
rs10419627	A	G	TRUE	0.5874	4.00E-04	5.00E-04	0.399	459184	0.6049	-0.0017	3.00E-04	3.20E-11	1195450	0.6047	0	5.00E-04	0.9485	852387
rs1042752	G	A		0.5428	-0.0032	5.00E-04	6.09E-12	460826	0.4399	-0.0018	3.00E-04	4.49E-12	1200940	0.5667	-0.0019	5.00E-04	8.76E-05	851687
rs10439970	G	T		0.684	-0.0035	5.00E-04	7.24E-13	460826	0.3178	-0.003	3.00E-04	2.42E-29	1198020	0.6829	0.0023	5.00E-04	6.94E-06	852671
rs10445262	A	G		0.7564	-0.0012	5.00E-04	0.02527	460826	0.6975	-0.0015	3.00E-04	1.89E-08	1196300	0.7	0.0012	6.00E-04	0.0276	851729
rs10447437	A	G		0.5477	-0.0014	5.00E-04	0.003152	460826	0.5325	-0.0015	3.00E-04	1.68E-09	1148220	0.5319	0.0011	5.00E-04	0.02803	800550
rs1047891	A	C	TRUE	0.3137	0.0039	5.00E-04	3.40E-15	460826	0.3044	-0.007	3.00E-04	4.91E-116	1158670	0.302	-0.0071	5.00E-04	2.77E-40	809561
rs1050816	C	T		0.3308	-0.001	5.00E-04	0.0365	460826	0.6703	-0.0031	3.00E-04	7.74E-32	1195800	0.334	9.00E-04	5.00E-04	0.08555	851228
rs10746942	G	A		0.6236	-0.0042	5.00E-04	1.04E-18	460826	0.3679	-0.0047	3.00E-04	1.64E-72	1187900	0.634	0.0034	5.00E-04	1.04E-11	838648
rs1075472	G	A		0.814	0.001	6.00E-04	0.09149	460826	0.1847	-0.0027	3.00E-04	6.63E-16	1035330	0.8133	0.0025	7.00E-04	0.000263	703536
rs10769264	T	C		0.385	-0.0026	5.00E-04	4.50E-08	460826	0.3726	-0.0023	3.00E-04	1.05E-19	1201300	0.3722	0.0035	5.00E-04	1.15E-12	852537
rs10790454	C	A		0.7322	-0.0021	5.00E-04	6.97E-05	460826	0.2608	-0.0021	3.00E-04	5.00E-15	1201870	0.7347	0.0034	6.00E-04	1.55E-09	852623
rs10797427	C	T		0.5946	-0.0021	5.00E-04	7.80E-06	460826	0.3956	-0.0017	3.00E-04	2.57E-11	1150150	0.6028	8.00E-04	5.00E-04	0.08547	804814
rs10821905	G	A		0.1794	-0.0041	6.00E-04	1.25E-11	460826	0.8201	-0.0043	3.00E-04	3.85E-37	1196300	0.1761	0.0025	7.00E-04	0.000185	851729
rs10821944	G	T		0.7265	-0.0014	5.00E-04	0.005799	460825	0.2882	-0.002	3.00E-04	1.95E-13	1199640	0.7109	0.0032	5.00E-04	9.12E-10	851605
rs10824855	G	A		0.4484	-0.0013	5.00E-04	0.005844	454011	0.5525	-0.0018	3.00E-04	1.76E-09	837675	0.45	3.00E-04	6.00E-04	0.6033	586451
rs1082710	T	G		0.3513	-0.0015	5.00E-04	0.002046	460826	0.3931	-0.0016	3.00E-04	2.21E-09	1201830	0.3949	-4.00E-04	5.00E-04	0.4286	852584
rs10827421	T	C		0.4639	-9.00E-04	5.00E-04	0.04414	460826	0.4439	-0.0018	3.00E-04	6.16E-12	1159620	0.4401	0.0013	5.00E-04	0.01106	810512
rs10850001	A	T		0.4315	-0.0088	5.00E-04	5.68E-78	460826	0.4535	-0.0017	3.00E-04	1.11E-10	1155920	0.4571	0.0016	5.00E-04	0.001572	808752
rs10851885	G	A		0.7488	-0.0065	5.00E-04	2.21E-34	460826	0.2485	-0.0056	3.00E-04	4.00E-69	1030720	0.7489	0.0092	6.00E-04	4.06E-51	700969
rs10857147	A	T		0.7112	-0.0029	5.00E-04	9.44E-09	460826	0.7039	-0.0033	3.00E-04	3.64E-36	1159620	0.7098	0.0029	5.00E-04	7.00E-08	810512
rs10866705	C	A		0.7551	-0.0079	5.00E-04	7.93E-49	460825	0.2606	-0.0063	3.00E-04	2.24E-120	1186370	0.7443	0.0053	6.00E-04	2.99E-21	843172
rs1087289	G	T		0.6034	-0.0028	5.00E-04	4.68E-09	460826	0.4121	-0.0018	3.00E-04	2.90E-12	1062490	0.5886	0.0023	5.00E-04	1.44E-05	768278
rs10878301	A	T	TRUE	0.7075	-9.00E-04	5.00E-04	0.07087	460826	0.6879	-0.0016	3.00E-04	4.59E-09	1201720	0.6751	-3.00E-04	5.00E-04	0.6075	852478
rs10892358	G	A		0.5547	-0.0012	5.00E-04	0.008208	460825	0.426	-0.0016	3.00E-04	2.13E-10	1148900	0.5741	4.00E-04	5.00E-04	0.4153	801003
rs10934753	A	G	TRUE	0.4139	1.00E-04	5.00E-04	0.8215	460826	0.4081	-0.002	3.00E-04	4.92E-15	1201920	0.4021	3.00E-04	5.00E-04	0.6009	852675
rs11057413	A	G		0.6674	-0.0015	5.00E-04	0.002001	460826	0.6759	-0.0018	3.00E-04	1.93E-09	909512	0.6797	-4.00E-04	6.00E-04	0.443	755581
rs11062102	C	T		0.3477	-5.00E-04	5.00E-04	0.2865	460826	0.6202	-0.004	3.00E-04	9.39E-53	1201680	0.388	0.0019	5.00E-04	0.000198	852430
rs11063202	G	A		0.8799	-0.0015	7.00E-04	0.02903	460826	0.1214	-0.0026	4.00E-04	3.19E-10	1200960	0.8781	0.0014	8.00E-04	0.07854	852296
rs11071738	T	C		0.5324	-0.0038	5.00E-04	3.04E-16	460826	0.5497	-0.0025	3.00E-04	1.59E-23	1201590	0.5563	0.0022	5.00E-04	4.67E-06	852344
rs11080660	A	G	TRUE	0.5577	-7.00E-04	5.00E-04	0.1436	460826	0.553	-0.0014	3.00E-04	3.63E-08	1159120	0.558	2.00E-04	5.00E-04	0.6321	810011
rs11109717	T	C		0.6977	-0.0025	5.00E-04	5.03E-07	460826	0.706	-0.0017	3.00E-04	2.71E-08	1199130	0.7124	0.002	5.00E-04	0.000246	852461
rs1111571	G	A	TRUE	0.7194	-6.00E-04	5.00E-04	0.2403	460826	0.2762	-0.0026	3.00E-04	6.71E-22	1198390	0.7288	-6.00E-04	5.00E-04	0.2553	850363
rs11123169	C	T		0.6683	-0.0029	5.00E-04	3.73E-09	460826	0.3253	-0.0025	3.00E-04	2.73E-21	1195190	0.6775	0.0063	5.00E-04	2.09E-35	850634
rs11160318	A	G		0.3188	-0.0022	5.00E-04	5.33E-06	460826	0.3649	-0.0017	3.00E-04	2.39E-10	1152940	0.3639	0.0014	5.00E-04	0.00878	809561
rs11170624	G	T		0.172	-0.0017	6.00E-04	0.005417	460826	0.8149	-0.0024	3.00E-04	2.32E-12	1154640	0.1901	8.00E-04	6.00E-04	0.2091	849346
rs11176071	A	G	TRUE	0.1331	-2.00E-04	7.00E-04	0.7196	451330	0.1322	-0.0023	4.00E-04	5.35E-09	1088820	0.1304	1.00E-04	8.00E-04	0.8781	765490
rs11202328	C	T	TRUE	0.1355	-8.00E-04	7.00E-04	0.2352	460826	0.786	-0.0022	4.00E-04	5.89E-10	1201580	0.2212	-1.00E-04	7.00E-04	0.9177	852333
rs11217554	T	C	TRUE	0.6294	4.00E-04	5.00E-04	0.3619	460826	0.6386	-0.0019	3.00E-04	7.32E-14	1201910	0.6409	2.00E-04	5.00E-04	0.705	852658
rs11237450	C	A		0.1653	0.0013	6.00E-04	0.04028	460826	0.789	-0.0026	3.00E-04	1.18E-14	1062090	0.2274	0.0017	6.00E-04	0.008425	768386
rs11243145	A	G		0.6179	-0.0041	5.00E-04	1.57E-17	460826	0.6261	-0.003	3.00E-04	1.28E-23	1004250	0.6241	0.0031	5.00E-04	1.10E-08	668751
rs11290509	T	C		0.0155	-0.0104	0.0019	4.76E-08	460826	0.0168	-0.0087	0.0012	2.85E-13	938492	0.0159	0.0116	0.0022	2.25E-07	644673
rs11344103	T	C		0.1669	-0.006	6.00E-04	1.48E-22	460826	0.1644	-0.0031	3.00E-04	9.73E-20	1201690	0.1655	0.0031	6.00E-04	1.34E-06	852445
rs11557049	T	C		0.0655	-0.005	9.00E-04	9.30E-08	451330	0.0645	-0.0044	6.00E-04	8.97E-14	928656	0.0656	0.0067	0.0011	1.24E-09	660968
rs11581477	A	G		0.0121	-0.0078	0.0021	0.000205	458306	0.0131	-0.0083	0.0013	4.22E-11	1011490	0.0122	0.0031	0.0024	0.206	686211
rs11613538	T	G		0.7817	-0.0021	6.00E-04	0.00017	460826	0.7679	-0.0019	3.00E-04	2.96E-09	1159620	0.761	0.001	6.00E-04	0.0838	810512
rs11616030	A	C		0.9158	-0.0048	8.00E-04	5.91E-09	460826	0.9119	-0.0039	5.00E-04	2.90E-16	994345	0.9126	0.0056	0.001	4.47E-09	658845
rs11646443	G	A	TRUE	0.3352	0	5.00E-04	0.9527	460826	0.6653	-0.0017	3.00E-04	1.68E-08	1118420	0.3259	-4.00E-04	6.00E-04	0.4501	785449
rs11673381	G	A		0.4733	-0.0015	5.00E-04	0.000848	459015	0.5081	-0.0014	3.00E-04	4.97E-08	1111820	0.4911	9.00E-04	5.00E-04	0.09743	776065
rs11676298	C	G		0.8117	-0.0017	6.00E-04	0.004369	460826	0.8144	-0.0023	3.00E-04	3.53E-12	1197380	0.8161	3.00E-04	6.00E-04	0.6854	852657
rs11725397	C	G	TRUE	0.2987	-6.00E-04	5.00E-04	0.2199	460826	0.3385	-0.0016	3.00E-04	1.28E-09	1200790	0.3467	2.00E-04	5.00E-04	0.635	852672
rs11743174	C	T		0.7297	-0.0022	5.00E-04	3.62E-05	460826	0.3046	-0.0018	3.00E-04	7.45E-11	1153280	0.6958	0.0017	6.00E-04	0.002942	804338
rs11745891	C	T		0.8948	-0.0055	7.00E-04	8.01E-14	460826	0.1085	-0.0056	4.00E-04	8.63E-41	1042260	0.892	0.0048	8.00E-04	1.06E-08	703941
rs11768336	T	C		0.2595	-0.0041	5.00E-04	4.45E-15	460826	0.3245	-0.0022	3.00E-04	1.13E-16	1159620	0.3264	1.00E-04	6.00E-04	0.9054	810512
rs11773903	T	G		0.0356	-0.0042	0.0013	0.000896	449148	0.0375	-0.0053	8.00E-04	2.77E-11	898624	0.0359	8.00E-04	0.0015	0.5724	638293
rs11786896	T	C	TRUE	0.0498	-8.00E-04	0.0011	0.4333	451330	0.0494	-0.006	7.00E-04	2.88E-19	958860	0.0494	0.0022	0.0013	0.07509	658270
rs11791149	C	T		0.8134	-0.0018	6.00E-04	0.002269	460826	0.207	-0.002	3.00E-04	1.96E-09	1153890	0.7767	-5.00E-04	6.00E-04	0.3593	810512
rs1183394	G	A		0.1167	-0.0032	7.00E-04	6.97E-06	460825	0.8715	-0.0024	4.00E-04	4.14E-09	1164050	0.1318	0.0019	7.00E-04	0.00925	827294
rs11856921	C	A		0.4251	-0.0035	5.00E-04	3.92E-14	460826	0.5867	-0.0027	3.00E-04	3.19E-25	1158520	0.4088	0.0017	5.00E-04	0.000626	809417
rs12024377	G	A		0.371	-0.0018	5.00E-04	0.00015	460826	0.5964	-0.0017	3.00E-04	1.85E-11	1159620	0.4065	0.0021	5.00E-04	8.59E-05	810512
rs12037201	T	G		0.2473	-0.0033	5.00E-04	4.55E-10	460826	0.2548	-0.0024	3.00E-04	4.49E-19	1200730	0.2563	7.00E-04	6.00E-04	0.2038	851477
rs12061708	A	G		0.3102	-0.0014	5.00E-04	0.005285	460826	0.2999	-0.0022	3.00E-04	2.74E-16	1197250	0.2968	8.00E-04	5.00E-04	0.1566	852680
rs12148121	C	T	TRUE	0.7662	-1.00E-04	5.00E-04	0.8918	460826	0.2748	-0.0018	3.00E-04	2.60E-11	1200950	0.7213	3.00E-04	6.00E-04	0.6332	851700
rs12148280	G	A		0.1005	-0.0051	8.00E-04	2.75E-11	460825	0.8947	-0.0028	4.00E-04	1.13E-10	1195800	0.1087	0.0028	8.00E-04	0.000467	851228
rs12152266	T	C	TRUE	0.2757	-1.00E-04	5.00E-04	0.8934	460826	0.308	-0.0019	3.00E-04	3.96E-12	1158180	0.3092	4.00E-04	6.00E-04	0.4199	810512
rs12212034	T	C		0.3479	-0.0033	5.00E-04	1.10E-11	460826	0.3606	-0.0022	3.00E-04	4.08E-17	1158180	0.3621	-7.00E-04	5.00E-04	0.1398	810512
rs12432201	C	T		0.6941	-0.0011	5.00E-04	0.02769	456193	0.3374	-0.002	3.00E-04	9.74E-15	1132600	0.6617	0.0014	5.00E-04	0.01235	791039
rs12436956	T	G		0.287	1.00E-04	5.00E-04	0.8863	460826	0.323	-0.0015	3.00E-04	1.53E-08	1153890	0.3281	0.002	5.00E-04	9.21E-05	810512
rs12439429	C	T	TRUE	0.5349	-5.00E-04	5.00E-04	0.2887	460826	0.4513	-0.0015	3.00E-04	2.59E-09	1201750	0.5496	7.00E-04	5.00E-04	0.1555	852498
rs12448902	C	G	TRUE	0.6095	0.0016	5.00E-04	0.000703	457835	0.6184	-0.0017	3.00E-04	2.47E-11	1201640	0.6241	-8.00E-04	5.00E-04	0.1169	852392
rs12449763	A	T		0.7765	-0.0014	6.00E-04	0.01512	460826	0.7876	-0.0022	3.00E-04	2.92E-11	1159620	0.787	0.0025	6.00E-04	9.24E-05	810512
rs12450700	T	G		0.6028	-0.0011	5.00E-04	0.01451	460826	0.6105	-0.0017	3.00E-04	2.83E-11	1159620	0.6119	0.0018	5.00E-04	0.000215	810512
rs12458009	T	G	TRUE	0.7703	8.00E-04	6.00E-04	0.1492	460825	0.7533	-0.0029	3.00E-04	1.51E-25	1192520	0.7473	-0.0022	6.00E-04	0.000149	849165
rs12483377	A	G		0.0869	-0.0032	8.00E-04	8.29E-05	459014	0.0852	-0.0028	5.00E-04	4.57E-09	1148240	0.086	0.0031	9.00E-04	0.001141	818247
rs12544197	A	G	TRUE	0.4925	-1.00E-04	5.00E-04	0.8045	460826	0.4909	-0.0016	3.00E-04	3.69E-10	1155360	0.4953	-2.00E-04	5.00E-04	0.7457	807478
rs12575164	T	C		0.7833	-4.00E-04	6.00E-04	0.4182	460826	0.7849	-0.0021	3.00E-04	1.91E-10	1201410	0.7914	0.002	6.00E-04	0.001057	852163
rs1258179	A	G		0.4904	-0.0018	5.00E-04	6.54E-05	460826	0.5065	-0.002	3.00E-04	9.76E-16	1201750	0.5077	-2.00E-04	5.00E-04	0.6543	852503

rs1260326	C	T		0.3949	-0.004	5.00E-04	2.81E-17	460824	0.5909	-0.005	3.00E-04	3.12E-84	1150400	0.4185	-0.0053	5.00E-04	1.01E-26	807178
rs12614953	T	C		0.5139	-0.0022	5.00E-04	1.43E-06	460826	0.4957	-0.0026	3.00E-04	4.79E-24	1159620	0.4919	4.00E-04	5.00E-04	0.4785	810512
rs12713261	C	T		0.6476	-0.0031	5.00E-04	9.84E-11	460826	0.3402	-0.0024	3.00E-04	8.03E-20	1159620	0.6552	-8.00E-04	5.00E-04	0.1465	810512
rs12727107	G	A		0.5472	-0.0012	5.00E-04	0.00997	460826	0.4717	-0.0014	3.00E-04	2.07E-08	1200980	0.5252	2.00E-04	5.00E-04	0.6702	851729
rs12736457	G	C	TRUE	0.8736	-1.00E-04	7.00E-04	0.917	460826	0.1295	-0.0057	4.00E-04	4.86E-45	1031540	0.8707	9.00E-04	8.00E-04	0.2334	699283
rs1275609	G	A		0.3269	-0.0015	5.00E-04	0.002603	460826	0.6385	-0.002	3.00E-04	1.18E-14	1158670	0.3634	0.0036	5.00E-04	3.24E-11	809561
rs12826808	A	T		0.7938	-0.0055	6.00E-04	5.68E-22	460826	0.7781	-0.0038	3.00E-04	4.54E-31	1201640	0.7665	0.0044	6.00E-04	1.04E-13	852385
rs12894354	T	C		0.3526	-0.001	5.00E-04	0.0338	460826	0.3468	-0.0017	3.00E-04	2.98E-11	1150630	0.3425	0.0018	5.00E-04	0.000473	810512
rs12907511	G	C		0.241	-0.0023	5.00E-04	2.91E-05	460826	0.7128	-0.0018	3.00E-04	1.35E-10	1159620	0.2871	0.0013	6.00E-04	0.02418	810512
rs1294861	G	T		0.7954	-0.0013	6.00E-04	0.02458	460826	0.2549	-0.0018	3.00E-04	4.14E-08	1201870	0.7239	0.0013	6.00E-04	0.02725	852625
rs12950549	G	A		0.6511	-0.0013	5.00E-04	0.00534	460826	0.3664	-0.0016	3.00E-04	1.39E-09	1201920	0.6335	0.0014	5.00E-04	0.004074	852675
rs13032786	G	C		0.7004	-0.0029	5.00E-04	1.28E-08	460826	0.3229	-0.003	3.00E-04	1.37E-29	1159620	0.6814	0.0026	5.00E-04	2.59E-06	810512
rs13059257	T	C		0.0291	-0.0046	0.0014	0.00069	459015	0.0294	-0.0053	8.00E-04	1.66E-10	974441	0.0298	0.01	0.0016	4.86E-10	644062
rs13064938	C	T	TRUE	0.3956	0	5.00E-04	0.9568	460826	0.5875	-0.0031	3.00E-04	1.22E-32	1201830	0.4157	6.00E-04	5.00E-04	0.212	852577
rs13108218	G	A	TRUE	0.387	-1.00E-04	5.00E-04	0.8173	457835	0.5955	-0.0018	3.00E-04	5.08E-12	1155350	0.4128	-3.00E-04	5.00E-04	0.6032	810011
rs13140982	G	A		0.287	-5.00E-04	5.00E-04	0.3596	460826	0.6607	-0.0016	3.00E-04	4.04E-09	1158670	0.3435	0.0025	6.00E-04	7.02E-06	809561
rs13157326	A	G		0.4624	-0.0021	5.00E-04	5.66E-06	460826	0.4573	-0.0024	3.00E-04	1.55E-21	1162800	0.451	0.0046	5.00E-04	3.61E-21	851618
rs13159523	A	G		0.513	-0.0024	5.00E-04	2.68E-07	459184	0.5028	-0.0025	3.00E-04	1.11E-22	1105130	0.5048	8.00E-04	5.00E-04	0.1132	791847
rs1316739	A	G		0.2337	-0.0021	5.00E-04	0.000155	460826	0.2738	-0.0016	3.00E-04	4.01E-09	1159620	0.2773	0.0023	6.00E-04	6.73E-05	810512
rs1321917	C	G		0.4094	-0.0021	5.00E-04	6.18E-06	460826	0.4259	-0.0023	3.00E-04	4.95E-19	1201890	0.4301	0.0015	5.00E-04	0.002284	852639
rs13245051	A	G		0.4546	-0.0016	5.00E-04	0.000372	460826	0.4518	-0.0019	3.00E-04	2.27E-13	1030500	0.4503	9.00E-04	5.00E-04	0.07881	689782
rs132639	A	T		0.18	-0.0017	6.00E-04	0.003774	460826	0.18	-0.0025	3.00E-04	8.83E-14	1193290	0.1832	0.0019	7.00E-04	0.006033	852069
rs1407040	C	T		0.6569	-0.0033	5.00E-04	9.80E-12	460826	0.3312	-0.002	3.00E-04	1.93E-14	1197420	0.6654	0.0029	5.00E-04	1.04E-08	852654
rs14863564	G	A		0.9123	-0.0049	8.00E-04	1.53E-09	460826	0.1008	-0.0038	4.00E-04	1.14E-18	1201930	0.9148	0.0045	9.00E-04	2.78E-07	852678
rs151245	T	G		0.5996	-0.0029	5.00E-04	1.05E-09	460826	0.6069	-0.0022	3.00E-04	9.61E-18	1178820	0.6102	0.0031	5.00E-04	3.01E-10	835458
rs1519845	G	A		0.6213	-7.00E-04	5.00E-04	0.1598	460826	0.3755	-0.0017	3.00E-04	1.64E-11	1196300	0.622	0.0014	5.00E-04	0.006902	851729
rs1541939	T	G		0.7328	-0.0013	5.00E-04	0.0129	460826	0.6803	-0.0029	3.00E-04	3.25E-20	1062240	0.6731	0.0024	6.00E-04	7.92E-05	768020
rs154656	A	T		0.4168	-0.0029	5.00E-04	8.16E-10	460826	0.4273	-0.0031	3.00E-04	1.73E-33	1193780	0.4226	-0.0017	5.00E-04	0.001252	852646
rs1548945	C	T		0.4202	-7.00E-04	5.00E-04	0.1274	460826	0.5696	-0.0037	3.00E-04	3.26E-47	1157170	0.4361	0.0014	5.00E-04	0.004606	810512
rs166775	T	C		0.7216	-0.0016	5.00E-04	0.0021	459184	0.6687	-0.0017	3.00E-04	1.65E-10	1148180	0.6676	0.0024	6.00E-04	1.01E-05	806468
rs16823029	A	C		0.0813	-0.0058	8.00E-04	4.81E-12	460826	0.0824	-0.0053	5.00E-04	1.08E-27	1039920	0.0833	0.0024	0.001	0.01006	704284
rs16827879	T	C		0.2399	-0.0017	5.00E-04	0.002064	460826	0.2387	-0.0018	3.00E-04	3.72E-08	1159620	0.2384	0.0017	6.00E-04	0.003325	810512
rs16874052	A	G		0.9519	-0.0062	0.0011	1.17E-08	460826	0.9512	-0.0044	6.00E-04	1.34E-13	1201880	0.9516	0.0064	0.0011	1.64E-08	852635
rs16930370	C	T		0.8267	-0.0012	6.00E-04	0.04197	460826	0.1713	-0.0042	3.00E-04	1.36E-34	1196300	0.8283	0.001	6.00E-04	0.1232	851729
rs17050272	A	G		0.4082	-0.0046	5.00E-04	2.89E-22	460826	0.4264	-0.0028	3.00E-04	7.08E-28	1190890	0.4181	0.0042	5.00E-04	3.08E-17	851729
rs1711667	T	G		0.5856	-0.0022	5.00E-04	1.78E-06	460826	0.5974	-0.0017	3.00E-04	8.55E-11	1196300	0.6062	3.00E-04	5.00E-04	0.5406	851729
rs1713842	T	A		0.1442	-0.0033	7.00E-04	5.01E-07	460826	0.8579	-0.0024	3.00E-04	1.68E-11	1183200	0.1387	0.0037	7.00E-04	1.45E-07	845094
rs1719935	G	A		0.5304	-0.0012	5.00E-04	0.007032	460826	0.451	-0.0026	3.00E-04	4.08E-24	1201720	0.5554	0	5.00E-04	0.9758	852474
rs17216707	T	C		0.8119	-0.0066	6.00E-04	2.69E-28	460826	0.81	-0.0048	3.00E-04	6.88E-46	1146580	0.8174	0.0065	7.00E-04	7.71E-23	801991
rs1741177	A	G	TRUE	0.6917	-9.00E-04	5.00E-04	0.08729	460826	0.7031	-0.0016	3.00E-04	8.82E-10	1156030	0.6997	0.001	5.00E-04	0.05375	808129
rs17413465	C	A		0.1854	-0.0018	6.00E-04	0.002546	460826	0.8155	-0.0025	3.00E-04	1.15E-13	1039940	0.1864	0.0026	7.00E-04	0.000132	704297
rs17420882	G	T		0.7238	-0.0035	5.00E-04	1.16E-11	460826	0.2855	-0.0024	3.00E-04	5.06E-20	1196300	0.7127	0.0028	5.00E-04	2.24E-07	851729
rs17563	G	A		0.4365	-0.0013	5.00E-04	0.00476	460826	0.5378	-0.0017	3.00E-04	4.29E-11	1153490	0.4679	0.0031	5.00E-04	2.40E-09	809060
rs1757915	G	A		0.3546	-0.0027	5.00E-04	1.11E-08	460825	0.6587	-0.0023	3.00E-04	1.66E-19	1192700	0.3428	0.0024	5.00E-04	6.86E-06	849346
rs17602729	G	A		0.1245	-0.0014	7.00E-04	0.04367	460826	0.8712	-0.0028	4.00E-04	2.34E-10	996165	0.1286	2.00E-04	8.00E-04	0.7701	703273
rs17638807	C	T	TRUE	0.4555	-7.00E-04	5.00E-04	0.1617	460826	0.5238	-0.0016	3.00E-04	6.49E-10	1201590	0.4817	7.00E-04	5.00E-04	0.1303	852342
rs17713396	T	C		0.3443	-0.004	5.00E-04	4.99E-17	460826	0.338	-0.0022	3.00E-04	1.61E-17	1200810	0.3317	0.0048	5.00E-04	2.63E-21	851570
rs1772976	C	T		0.5148	-9.00E-04	5.00E-04	0.05117	460826	0.5087	-0.0016	3.00E-04	1.42E-10	1159620	0.4851	0.001	5.00E-04	0.03384	810512
rs17730281	G	A		0.2263	-0.0051	5.00E-04	1.93E-20	460826	0.747	-0.0043	3.00E-04	2.44E-54	1196300	0.2527	0.006	6.00E-04	1.53E-25	851729
rs1783827	A	G		0.563	-0.0013	5.00E-04	0.005383	460826	0.5799	-0.0019	3.00E-04	2.03E-13	1159620	0.5818	0	5.00E-04	0.983	810512
rs1800574	T	C		0.0287	-0.0031	0.0014	0.02351	460826	0.031	-0.0065	8.00E-04	8.82E-16	1026470	0.0307	0.0031	0.0016	0.04724	696461
rs1801251	G	A		0.3605	-0.0028	5.00E-04	6.50E-09	460826	0.6497	-0.0019	3.00E-04	7.73E-14	1192700	0.3534	0.0018	5.00E-04	0.000412	849339
rs1844334	C	T		0.3439	-8.00E-04	5.00E-04	0.08774	460826	0.6346	-0.002	3.00E-04	2.55E-14	1201880	0.3692	0.0016	5.00E-04	0.003462	852627
rs1851285	G	C		0.5253	-0.0019	5.00E-04	3.49E-05	460826	0.4933	-0.0015	3.00E-04	2.58E-09	1159620	0.499	0.0014	5.00E-04	0.004424	810512
rs18553810	A	G	TRUE	0.9891	0.0029	0.0023	0.2114	436765	0.9895	-0.0086	0.0014	1.34E-09	959897	0.9893	0.002	0.0027	0.4621	637372
rs1857859	G	A		0.2706	-0.001	5.00E-04	0.0512	460826	0.7062	-0.0018	3.00E-04	6.33E-11	1191260	0.2979	0.0025	5.00E-04	1.36E-06	849346
rs1858800	C	T		0.3403	-0.0018	5.00E-04	0.000242	460826	0.6694	-0.0022	3.00E-04	7.09E-18	1191750	0.3318	-0.0012	5.00E-04	0.02154	848392
rs18735570	G	C		0.9752	-0.0141	0.0015	6.19E-22	460826	0.0276	-0.0116	9.00E-04	4.16E-40	980431	0.9732	0.0155	0.0017	1.22E-19	649909
rs1887252	C	G		0.6358	-0.0032	5.00E-04	1.77E-11	460826	0.6263	-0.0024	3.00E-04	6.08E-20	1201560	0.6239	0.0044	5.00E-04	1.31E-18	852310
rs1913641	T	G		0.4791	-0.0012	5.00E-04	0.006617	460826	0.4907	-0.0019	3.00E-04	2.78E-14	1201850	0.4922	7.00E-04	5.00E-04	0.1282	852601
rs2007458	A	G	TRUE	0.5906	1.00E-04	5.00E-04	0.8257	460826	0.5688	-0.0021	3.00E-04	8.90E-17	1201910	0.5575	-6.00E-04	5.00E-04	0.2272	852664
rs2034899	G	T		0.7158	-0.0013	5.00E-04	0.009733	460826	0.2755	-0.002	3.00E-04	6.94E-14	1201880	0.7219	0.0011	5.00E-04	0.04185	852631
rs2055873	C	T		0.299	-0.0016	5.00E-04	0.002059	460825	0.7058	-0.0015	3.00E-04	1.01E-08	1185130	0.3002	0.001	5.00E-04	0.04768	841776
rs2068888	A	G		0.4524	-0.0036	5.00E-04	1.45E-14	460826	0.4722	-0.0029	3.00E-04	2.59E-30	1192700	0.4753	0.0027	5.00E-04	2.31E-08	849346
rs2072379	T	C		0.6752	-0.0017	5.00E-04	0.000737	459184	0.6878	-0.0021	3.00E-04	3.14E-16	1151060	0.6859	8.00E-04	5.00E-04	0.1203	808573
rs2074292	G	A		0.5497	-0.0018	5.00E-04	7.23E-05	460825	0.455	-0.0015	3.00E-04	9.59E-09	1119360	0.5464	8.00E-04	5.00E-04	0.1122	809524
rs2076668	A	G		0.3754	-8.00E-04	5.00E-04	0.08408	460826	0.3687	-0.0037	3.00E-04	1.32E-46	1159130	0.3617	0.0014	5.00E-04	0.005701	810512
rs2095567	C	T		0.7642	-0.002	5.00E-04	0.000213	460826	0.2686	-0.0018	3.00E-04	1.73E-11	1159620	0.734	0	6.00E-04	0.9924	810512
rs2096551	C	G		0.3941	-0.002	5.00E-04	3.09E-05	460826	0.3962	-0.0016	3.00E-04	2.57E-10	1201600	0.395	8.00E-04	5.00E-04	0.1002	852350
rs2158812	T	C	TRUE	0.0257	-0.0023	0.0015	0.1251	460826	0.0709	-0.0052	7.00E-04	1.60E-14	1191940	0.071	-5.00E-04	0.0013	0.699	843660
rs216364	C	T		0.7283	0	5.00E-04	0.9295	460826	0.2701	-0.0021	3.00E-04	4.03E-15	1192700	0.7258	0.0017	6.00E-04	0.002009	849346
rs2185786	T	C	TRUE	0.6095	-5.00E-04	5.00E-04	0.2622	460826	0.6027	-0.0015	3.00E-04	2.29E-09	1201830	0.6035	5.00E-04	5.00E-04	0.347	852585
rs2186797	C	T		0.9339	6.00E-04	9.00E-04	0.4925	460826	0.064	-0.0031	5.00E-04	1.70E-08	977082	0.9318	0.0028	0.0011	0.009533	653516
rs219786	T	C		0.7855	-0.0032	6.00E-04	9.49E-09	460826	0.7871	-0.0026	3.00E-04	2.39E-15	1038970	0.7862	0.0066	6.00E-04	2.55E-25	703334
rs223317	T	C		0.6651	-0.0043	5.00E-04	7.73E-19	460826	0.6634	-0.003	3.00E-04	8.74E-30	1201920	0.6601	0.0033	5.00E-04	4.08E-11	852675
rs2235826	A	T		0.8151	-0.0021	6.00E-04	0.000557	460826	0.8012	-0.0031	3.00E-04	1.18E-20	1158520	0.7997	0.0024	6.00E-04	0.000132	809417
rs2236520	T	C		0.6891	-0.0018	5.00E-04	0.000433	457835	0.7006	-0.0021	3.00E-04	6.86E-15	1159620	0.7001	0.0013	5.00E-04	0.01985	810512
rs2241358	C	G		0.6724	-0.0027	5.00E-04	3.53E-08	460826	0.6868	-0.0016	3.00E-04	6.16E-10	1144310	0.6844	-0.0019	5.00E-04	0.000383	806825
rs2250067	T	C		0.4759	-0.0036	5.00E-04	2.54E-15	460826	0.4839	-0.0028	3.00E-04	2.07E-28	1159620	0.4945	0.005	5.00E-04	8.81E-25	810512
rs2274786	C	T	TRUE	0.6515	-9.00E-04	5.00E-04	0.06549	460826	0.3546	-0.0015	3.00E-04	2.79E-09	1158520	0.6427	-4.00E-04	5.00E-04	0.3918	809417
rs2275848	G	T		0.8122	-0.0013	6.00E-04	0.02596	460826	0.1886	-0.0021	3.00E-04	5.04E-10	1132270	0.8119	3.00E-04	7.00E-04	0.597	789278
rs227731	G	T	TRUE	0.5417	-8.00E-04	5.00E-04	0.07694	460826	0.4423	-0.0021	3.00E-04	3.53E-16	1153990	0.5583	1.00E-04	5.00E-04	0.7927	809561
rs2277383	G	T		0.8857	-0.0022	7.00E-04	0.002825	460824	0.1552	-0.0025	4.00E-04	1.74E-11	1192700	0.842	0.0034	7.00E-04	1.45E-06	849346
rs2279252	G	A		0.0127	-0.0068	0.0021	0.001092	459184	0.9505	-0.0052	9.00E-04	4.82E-09	1089870	0.0566	0.003	0.0017	0.07245	762128
rs2289746	T	C		0.3372	-9.00E-04	5.00E-04	0.06816	460826	0.377	-0.0019	3.00E-04	1.43E-12	1196280	0.3845	0.0011	5.00E-04	0.03744	851717
rs2293605	C	T		0.1183	-0.0019	7.00E-04	0.00737	460825	0.8797	-0.0024	4.00E-04	4.59E-09	1134700	0.1204	6.00E-04	8.00E-04	0.4314	790907
rs2297129	A	G		0.6635	-0.0017	5.00E-04	0.000509	460826	0.6621	-0.0022	3.00E-04	3.43E-17	1158230	0.6627	0.0011	5.00E-04	0.02579	851729
rs2297853	G	T	TRUE	0.3525	2.00E-04	5.00E-04	0.6287	460826	0.6314	-0.0015	3.00E-04	8.67E-09	1200260	0.37	-5.00E-04	5.00E-04	0.3624	852462
rs2301343	T	G	TRUE	0.756	-9.00E-04	5.00E-04	0.0792	460826	0.7565	-0.0024	3.00E-04	2.11E-14	1192700	0.7631	9.00E-04	6.00E-04	0.1411	849346
rs2306623	T	C		0.3333	-0.0034	5.00E-04	5.78E-12	460826	0.32	-0.002	3.00E-04	6.66E-15	1158670	0.3133	-7.00E-04	5.00E-04	0.2224	790268
rs2338796	G	A		0.6726	-0.0019	5.00E-04	9.87E-05	456193	0.3297	-0.0046	3.00E-04	4.16E-70	1177720	0.6781	-1.00E-04	5.00E-04	0.8508	849603
rs2346418	G	A		0.5124	-0.0013	5.00E-04	0.003778	460826	0.5015	-0.0016	3.00E-04	1.45E-10	1158000	0.4959	0.0014	5.00E-04	0.005775	808895
rs2381199	G	T		0.8047	-0.0018	6.00E-04	0.002601	436765	0.2547	-0.0019	3.00E-04	1.79E-08	834255	0.7427	4.00E-04	6.00E-04	0.5344	675600
rs2412608	C	T		0.4901	-0.0029	5.00E-04	3.53E-10	460826	0.5065	-0.0033	3.00E-04	1.51E-38	1002100	0.4894	4.00E-04	5.00E-04	0.4811	663016
rs243067	G	T		0.398	-0.0017	5.00E-04	0.000287	460826	0.6037	-0.0014	3.00E-04	1.74E-08	1049430	0.399	5.00E-04	5.00E-04	0.3423	704184
rs2433601	C	T	TRUE	0.6163	-1.00E-04	5.00E-04	0.7901	460826	0.3982	-0.009	3.00E-04	1.13E-262	1201820	0.6005	-7.00E-04	5.00E-04	0.1729	852567
rs2440164	G	A	TRUE	0.6162	-8.00E-04	5.00E-04	0.07652	460826	0.3721	-0.0041	3.00E-04	3.24E-57	1154210	0.6298	9.00E-04	5.00E-04	0.09367	810512

rs246234	G	C	TRUE	0.305	0.001	5.00E-04	0.0562	460826	0.6988	-0.0016	3.00E-04	2.19E-09	1201730	0.3016	0.001	5.00E-04	0.05489	852484
rs2469953	T	C		0.4005	-3.00E-04	5.00E-04	0.4861	460826	0.4383	-0.0015	3.00E-04	1.45E-08	1158230	0.4331	0.0021	5.00E-04	1.66E-05	851729
rs2515414	C	A	TRUE	0.4798	-3.00E-04	5.00E-04	0.5312	460826	0.5396	-0.0014	3.00E-04	2.55E-08	1159620	0.4587	-3.00E-04	5.00E-04	0.5356	810512
rs2551644	A	T	TRUE	0.8166	-8.00E-04	6.00E-04	0.1958	460826	0.8068	-0.0019	3.00E-04	2.75E-08	996356	0.8099	0	7.00E-04	0.9455	662135
rs2577134	C	T	TRUE	0.7508	-6.00E-04	5.00E-04	0.277	460826	0.2902	-0.0015	3.00E-04	2.13E-08	1159620	0.7041	0.001	6.00E-04	0.08488	810512
rs2607775	G	C		0.517	-0.0015	5.00E-04	0.001229	460826	0.479	-0.0015	3.00E-04	3.82E-09	1201900	0.5241	8.00E-04	5.00E-04	0.1128	852646
rs2625166	G	A		0.4313	-0.0011	5.00E-04	0.02345	436765	0.5583	-0.0014	3.00E-04	3.43E-08	1061440	0.4416	0.0016	5.00E-04	0.002798	767225
rs2634675	G	A	TRUE	0.4561	-3.00E-04	5.00E-04	0.4531	460826	0.5133	-0.0024	3.00E-04	3.24E-20	1162680	0.4853	5.00E-04	5.00E-04	0.3362	851501
rs264608	C	T		0.2669	-0.0023	5.00E-04	1.03E-05	460826	0.6903	-0.0018	3.00E-04	3.63E-11	1015920	0.3109	0.0029	6.00E-04	2.67E-07	802092
rs267738	T	G		0.7853	-0.0053	6.00E-04	1.39E-21	460826	0.7901	-0.0051	3.00E-04	8.31E-57	1192500	0.7902	0.0026	6.00E-04	3.79E-05	849148
rs27028	A	G		0.3715	-0.0019	5.00E-04	6.21E-05	460826	0.3592	-0.0016	3.00E-04	6.45E-10	1199850	0.3577	8.00E-04	5.00E-04	0.1088	850616
rs2720526	G	A	TRUE	0.7432	-3.00E-04	5.00E-04	0.601	460826	0.253	-0.0015	3.00E-04	1.98E-08	1201600	0.7496	-1.00E-04	6.00E-04	0.8181	852353
rs2749153	A	G	TRUE	0.7322	-8.00E-04	5.00E-04	0.1099	460826	0.7093	-0.003	3.00E-04	5.67E-28	1200920	0.7015	0.001	5.00E-04	0.05143	851673
rs2780955	A	T		0.4115	-0.001	5.00E-04	0.03438	460826	0.3962	-0.0014	3.00E-04	3.76E-08	1157870	0.3962	3.00E-04	5.00E-04	0.54	809558
rs2781649	G	A	TRUE	0.7736	-7.00E-04	6.00E-04	0.2377	460826	0.241	-0.003	3.00E-04	6.20E-21	1120110	0.7567	-0.0015	6.00E-04	0.007398	810512
rs2808454	T	A		0.4569	-0.0021	5.00E-04	5.55E-06	460826	0.5196	-0.0021	3.00E-04	4.13E-16	1201560	0.4872	0.0019	5.00E-04	0.000116	852309
rs281380	T	C		0.6349	-0.0022	5.00E-04	5.70E-06	460825	0.5954	-0.0021	3.00E-04	8.72E-16	1151060	0.5966	-0.0047	5.00E-04	5.15E-19	808573
rs2815374	A	G		0.6507	-0.0041	5.00E-04	3.78E-17	460826	0.6614	-0.0018	3.00E-04	1.41E-11	1159620	0.6649	0.0052	5.00E-04	1.34E-22	810512
rs2823139	A	G		0.3369	-0.0038	5.00E-04	7.46E-15	460825	0.3332	-0.0031	3.00E-04	7.20E-32	1192740	0.3298	0.0045	5.00E-04	5.04E-19	850634
rs2834320	G	A		0.8385	-0.0055	6.00E-04	2.06E-18	460826	0.1555	-0.0036	3.00E-04	3.68E-25	1200960	0.8425	0.0028	7.00E-04	3.74E-05	851715
rs284859	G	T		0.1733	-0.0019	6.00E-04	0.001391	460826	0.8065	-0.0029	3.00E-04	7.34E-18	1193350	0.1894	0.0013	6.00E-04	0.04938	849993
rs28490558	A	G		0.2445	-0.0018	5.00E-04	0.000554	456193	0.2491	-0.0021	3.00E-04	2.43E-11	1057600	0.246	0.0016	6.00E-04	0.01128	775317
rs2873542	G	T		0.9381	-0.0047	0.001	1.45E-06	460826	0.0711	-0.0033	5.00E-04	1.91E-10	1152640	0.9272	0.0017	0.001	0.09993	812583
rs2880624	C	T	TRUE	0.5936	2.00E-04	5.00E-04	0.747	460826	0.3986	-0.0014	3.00E-04	2.88E-08	1145740	0.6013	9.00E-04	5.00E-04	0.07927	796624
rs2881741	T	C		0.4552	-0.0115	5.00E-04	4.51E-132	436765	0.425	-0.0078	3.00E-04	2.41E-206	1201870	0.424	0.0076	5.00E-04	2.70E-54	852621
rs288753	G	A		0.3593	-0.0047	5.00E-04	3.30E-22	456193	0.6513	-0.0027	3.00E-04	2.79E-25	1138740	0.3453	0.0017	5.00E-04	0.001186	804313
rs2891028	C	T		0.326	-0.0015	5.00E-04	0.002761	460826	0.6939	-0.0029	3.00E-04	5.70E-29	1150880	0.3098	0.0022	5.00E-04	4.55E-05	808345
rs2906163	C	T	TRUE	0.6487	-9.00E-04	5.00E-04	0.07282	460826	0.3352	-0.0014	3.00E-04	2.81E-08	1153920	0.6616	0.001	5.00E-04	0.06978	804814
rs2953516	C	T		0.2416	-0.0023	5.00E-04	1.39E-05	460826	0.705	-0.0026	3.00E-04	3.59E-22	1201540	0.3027	7.00E-04	5.00E-04	0.2049	852288
rs2954017	C	T		0.4768	-0.002	5.00E-04	1.61E-05	460826	0.5308	-0.0029	3.00E-04	1.13E-30	1121560	0.4719	-0.0016	5.00E-04	0.000773	810512
rs2991341	T	C		0.4131	-0.0018	5.00E-04	7.81E-05	460826	0.3907	-0.0019	3.00E-04	9.90E-14	1159620	0.389	6.00E-04	5.00E-04	0.2616	810512
rs303937	T	A		0.4044	-0.0044	5.00E-04	3.47E-21	460826	0.5636	-0.0028	3.00E-04	2.73E-27	1159620	0.4396	0.0059	5.00E-04	4.38E-30	810512
rs3060	C	T		0.9057	-0.0025	8.00E-04	0.001856	460826	0.157	-0.0022	4.00E-04	8.35E-09	1192700	0.831	6.00E-04	7.00E-04	0.4165	849346
rs3111257	T	C		0.5613	-0.0013	5.00E-04	0.006421	460826	0.5622	-0.0015	3.00E-04	1.45E-08	1201870	0.5628	0.0024	5.00E-04	1.69E-06	852623
rs3119304	C	T	TRUE	0.8704	-7.00E-04	7.00E-04	0.3307	460826	0.1206	-0.0084	4.00E-04	1.07E-96	1158180	0.8756	5.00E-04	8.00E-04	0.5554	810512
rs3417403	A	G		0.0233	-0.0047	0.0015	0.002279	460826	0.023	-0.0055	0.001	1.95E-08	922953	0.0231	0.0053	0.0019	0.004449	631019
rs3418829	C	G		0.2694	-0.0018	5.00E-04	0.000692	451330	0.2832	-0.0017	3.00E-04	4.11E-08	1075140	0.2939	0.0018	6.00E-04	0.001646	753729
rs3429313	T	C		0.6936	-0.0028	5.00E-04	1.19E-08	460826	0.6963	-0.0024	3.00E-04	2.02E-19	1201860	0.7062	8.00E-04	5.00E-04	0.1356	852609
rs3435713	C	G		0.7358	-0.0025	5.00E-04	1.56E-06	460826	0.7315	-0.0028	3.00E-04	1.25E-25	1159620	0.7364	0.0021	6.00E-04	0.000129	810512
rs3444253	G	C		0.6825	-0.0027	5.00E-04	1.08E-07	460826	0.3151	-0.0019	3.00E-04	4.70E-13	1159120	0.6864	0	5.00E-04	0.9784	810011
rs3446033	T	C	TRUE	0.9211	-9.00E-04	8.00E-04	0.2674	460826	0.9149	-0.0034	5.00E-04	2.10E-12	1043920	0.9199	0	0.001	0.969	703352
rs3486176	T	C		0.4096	-0.0074	5.00E-04	2.65E-56	460826	0.4006	-0.0048	3.00E-04	7.42E-79	1201860	0.3911	0.0044	5.00E-04	2.12E-19	852608
rs3495002	G	A	TRUE	0.3166	5.00E-04	5.00E-04	0.3459	460826	0.6862	-0.0024	3.00E-04	1.11E-20	1158180	0.3155	-2.00E-04	5.00E-04	0.6547	810512
rs3507210	A	G		0.5347	-0.0014	5.00E-04	0.003053	460826	0.5569	-0.0019	3.00E-04	4.64E-14	1200870	0.554	0.0011	5.00E-04	0.03036	851618
rs3591557	C	T		0.405	-0.0028	5.00E-04	1.55E-09	460826	0.5907	-0.0022	3.00E-04	3.67E-17	1159620	0.4049	0.0021	5.00E-04	1.93E-05	810512
rs3596957	T	G		0.4087	-0.0035	5.00E-04	4.65E-14	460826	0.3946	-0.0056	3.00E-04	1.14E-106	1159620	0.3939	0.0022	5.00E-04	2.28E-05	810512
rs3750081	T	G		0.59	-0.0012	5.00E-04	0.00955	460826	0.5917	-0.0022	3.00E-04	6.60E-18	1159620	0.588	-1.00E-04	5.00E-04	0.857	810512
rs3755880	A	G		0.2856	-0.0021	5.00E-04	5.27E-05	460826	0.2878	-0.0015	3.00E-04	2.19E-08	1201590	0.2917	-1.00E-04	5.00E-04	0.7811	852342
rs3757387	C	T		0.552	-0.0063	5.00E-04	2.40E-42	460826	0.4301	-0.0027	3.00E-04	6.54E-27	1158180	0.5718	0.002	5.00E-04	0.000111	810512
rs3775932	A	C		0.4935	-9.00E-04	5.00E-04	0.04077	460826	0.5024	-0.0019	3.00E-04	3.25E-14	1159620	0.5002	9.00E-04	5.00E-04	0.08085	810512
rs3794991	T	C		0.0847	-0.0045	8.00E-04	5.43E-08	460826	0.0879	-0.0033	4.00E-04	5.85E-14	1201800	0.0893	1.00E-04	9.00E-04	0.9158	852556
rs3795503	C	T		0.3145	-0.0029	5.00E-04	7.61E-09	460826	0.6591	-0.0023	3.00E-04	3.31E-18	1157170	0.3358	0.0024	5.00E-04	7.98E-06	810512
rs3809627	C	A		0.4041	-0.0016	5.00E-04	0.000767	456193	0.5658	-0.0015	3.00E-04	1.27E-08	1157900	0.4392	6.00E-04	5.00E-04	0.2043	809561
rs3814995	C	T		0.3121	-0.0013	5.00E-04	0.01164	450809	0.6583	-0.0023	3.00E-04	3.43E-14	1106950	0.3573	0.0023	5.00E-04	2.63E-05	802502
rs3820201	A	G		0.6158	-8.00E-04	5.00E-04	0.1018	460825	0.5826	-0.0016	3.00E-04	5.81E-10	1153040	0.5752	0.0025	5.00E-04	1.67E-06	808607
rs3842761	G	C		0.2407	-0.0047	6.00E-04	2.47E-17	436765	0.7256	-0.0038	3.00E-04	5.64E-33	1134100	0.2878	0.0045	6.00E-04	4.52E-15	802777
rs3850625	G	A		0.1158	-0.0013	7.00E-04	0.07429	460826	0.881	-0.0049	4.00E-04	1.59E-32	1174580	0.1154	0.0022	8.00E-04	0.007637	831446
rs3918226	T	C		0.08	-0.0041	9.00E-04	1.39E-06	460826	0.0826	-0.0033	5.00E-04	2.51E-11	1017730	0.082	0.0015	0.001	0.1295	699833
rs3925003	T	C	TRUE	0.5697	-9.00E-04	5.00E-04	0.0569	460826	0.575	-0.0016	3.00E-04	1.11E-09	1191260	0.5778	9.00E-04	5.00E-04	0.0557	849346
rs4128481	T	G		0.0187	-0.0056	0.0017	0.001161	460826	0.0226	-0.0083	0.001	3.50E-17	971381	0.0223	-0.0029	0.0019	0.2265	648755
rs4130139	C	T		0.2824	-0.0015	5.00E-04	0.003203	460826	0.6951	-0.0027	3.00E-04	1.90E-23	1201770	0.3046	0.0029	5.00E-04	2.39E-08	852526
rs417237	G	T	TRUE	0.6132	-1.00E-04	5.00E-04	0.8776	460826	0.4091	-0.0017	3.00E-04	1.29E-10	1196300	0.5844	-2.00E-04	5.00E-04	0.6959	851729
rs4245230	A	G		0.4619	-0.0016	5.00E-04	0.000884	459184	0.4733	-0.0022	3.00E-04	3.81E-18	1156380	0.4671	4.00E-04	5.00E-04	0.4148	808041
rs4275190	T	C	TRUE	0.6977	0	5.00E-04	0.9748	460826	0.6936	-0.0018	3.00E-04	5.06E-12	1201180	0.6902	0	5.00E-04	0.9927	852416
rs429358	T	C		0.8456	-0.0056	6.00E-04	2.88E-18	459015	0.848	-0.0029	3.00E-04	3.00E-17	1141110	0.8506	0.0043	7.00E-04	9.05E-10	803659
rs4376843	C	G		0.2681	-0.0027	5.00E-04	3.49E-07	460826	0.31	-0.0017	3.00E-04	1.34E-10	1201850	0.3073	0.0037	6.00E-04	3.83E-11	852602
rs4442348	G	A		0.5016	-0.0028	5.00E-04	7.63E-10	460826	0.515	-0.0018	3.00E-04	7.54E-13	1194320	0.477	2.00E-04	5.00E-04	0.6452	845066
rs4491726	G	A		0.688	-0.0044	5.00E-04	2.64E-19	460826	0.3137	-0.0034	3.00E-04	1.20E-28	1201260	0.6888	0.0019	6.00E-04	0.000753	852598
rs4515954	C	A		0.829	-0.0025	6.00E-04	3.76E-05	460826	0.1919	-0.0023	3.00E-04	1.66E-11	1038850	0.8071	0.0014	7.00E-04	0.03811	768306
rs4561993	T	G		0.3123	-0.0029	5.00E-04	5.58E-09	460826	0.3404	-0.003	3.00E-04	1.11E-30	1159620	0.3426	0.002	5.00E-04	0.000251	810512
rs4666821	G	T		0.513	-0.0027	5.00E-04	5.02E-09	460826	0.4787	-0.002	3.00E-04	7.10E-15	1199260	0.5229	0.0027	5.00E-04	2.73E-08	852459
rs4683730	A	G		0.3671	-0.0017	5.00E-04	0.000449	460826	0.3641	-0.0014	3.00E-04	4.18E-08	1159620	0.3696	0.0019	5.00E-04	0.000325	810512
rs4837207	G	T		0.864	-0.0028	7.00E-04	5.48E-05	460826	0.2436	-0.0021	4.00E-04	7.37E-09	1153890	0.7291	0.0017	7.00E-04	0.01295	810512
rs4854645	A	G		0.4738	-0.0014	5.00E-04	0.002534	460826	0.4829	-0.0016	3.00E-04	1.33E-10	1201650	0.4861	1.00E-04	5.00E-04	0.8004	852404
rs4925095	A	G	TRUE	0.4991	0	5.00E-04	0.9914	460826	0.4869	-0.0019	3.00E-04	6.44E-14	1201840	0.4906	5.00E-04	5.00E-04	0.3224	852589
rs4970765	C	T		0.7061	-0.0014	5.00E-04	0.006438	460826	0.2976	-0.0031	3.00E-04	1.88E-24	1162620	0.7029	-9.00E-04	6.00E-04	0.1247	851436
rs4971092	T	C		0.8815	-0.0045	7.00E-04	3.11E-10	456193	0.8799	-0.0028	4.00E-04	6.52E-12	1054720	0.8799	0.0084	8.00E-04	1.35E-25	711511
rs5010824	G	A		0.147	-0.0011	7.00E-04	0.09931	460826	0.852	-0.0021	3.00E-04	2.27E-09	1201920	0.1475	0.0018	7.00E-04	0.007228	852672
rs550057	C	T	TRUE	0.253	1.00E-04	5.00E-04	0.785	460826	0.7421	-0.0023	3.00E-04	1.30E-17	1192940	0.2569	-0.0043	6.00E-04	9.03E-15	850639
rs5565848	G	A		0.3347	-0.0042	5.00E-04	6.62E-18	460826	0.6773	-0.0019	3.00E-04	5.94E-13	1199280	0.3211	0.0041	5.00E-04	1.32E-14	852486
rs5574302	T	G		0.6238	-0.0039	5.00E-04	8.65E-17	460826	0.6293	-0.0034	3.00E-04	1.23E-40	1201860	0.6282	0.0027	5.00E-04	3.60E-07	852614
rs5575461	C	T	TRUE	0.193	-8.00E-04	6.00E-04	0.2041	460826	0.8086	-0.0019	3.00E-04	1.07E-08	1159620	0.1917	7.00E-04	6.00E-04	0.2833	810512
rs5579762	T	C		0.7448	-0.0019	5.00E-04	0.000232	460826	0.7475	-0.0015	3.00E-04	2.10E-08	1201920	0.7462	3.00E-04	6.00E-04	0.6504	852666
rs5584228	A	G		0.3662	-0.0023	5.00E-04	1.50E-06	460826	0.4037	-0.0022	3.00E-04	1.34E-16	1159620	0.4095	4.00E-04	5.00E-04	0.4494	810512
rs5592491	G	C		0.8411	-0.003	6.00E-04	2.16E-06	460826	0.1541	-0.0024	3.00E-04	5.72E-12	1201630	0.8483	0.0022	7.00E-04	0.001526	852383
rs5603489	T	C		0.4689	-0.0021	5.00E-04	9.14E-06	456193	0.4533	-0.0016	3.00E-04	1.11E-09	1141240	0.4489	0.0014	5.00E-04	0.003593	802146
rs5606555	C	G		0.3119	-6.00E-04	5.00E-04	0.2344	460826	0.3166	-0.0031	3.00E-04	8.19E-32	1155720	0.3227	0.0024	5.00E-04	1.04E-05	810512
rs5626156	T	C		0.346	-0.0026	5.00E-04	1.16E-07	460826	0.3508	-0.0017	3.00E-04	1.98E-08	954536	0.3456	0.0011	6.00E-04	0.06158	656825
rs5628355	G	A	TRUE	0.0931	-9.00E-04	8.00E-04	0.2423	460826	0.856	-0.0053	4.00E-04	1.34E-44	1196170	0.1523	1.00E-04	7.00E-04	0.9021	852657
rs5630629	A	T	TRUE	0.2451	-5.00E-04	5.00E-04	0.3724	460826	0.2487	-0.0026	3.00E-04	5.31E-16	1062250	0.2425	1.00E-04	6.00E-04	0.8316	768038
rs5744566	G	A		0.2033	-0.0025	6.00E-04	1.33E-05	460826	0.7994	-0.0022	3.00E-04	1.48E-11	1159120	0.2008	0.0028	6.00E-04	7.29E-06	810011
rs5750561	T	A		0.4022	-0.002	5.00E-04	1.86E-05	460826	0.6051	-0.0024	3.00E-04	1.46E-20	1153890	0.3921	0.0012	5.00E-04	0.01846	810512
rs5760006	C	T		0.9014	-0.0021	8.00E-04	0.008379	460826	0.1784	-0.0021	4.00E-04	3.84E-08	1158670	0.8129	0.0018	7.00E-04	0.0127	809561
rs580114	A	G	TRUE	0.2944	-4.00E-04	5.00E-04	0.4441	460826	0.285	-0.0018	3.00E-04	3.45E-11	1201900	0.2835	1.00E-04	5.00E-04	0.926	852646
rs5811742	A	G		0.2314	-0.0024	5.00E-04	1.02E-05	460826	0.2281	-0.0019	3.00E-04	3.44E-09	1201810	0.2262	0.0022	6.00E-04	0.000206	852559
rs5934308	A	T		0.1454	-0.0012	7.00E-04	0.06644	460826	0.1421	-0.0025	3.00E-04	6.49E-13	1159620	0.1471	0.0019	7.00E-04	0.006079	810512
rs594672	T	C		0.5329	-0.0018	5.00E-04	0.000128	460826	0.5167	-0.0014	3.00E-04	1.77E-08	1196090	0.5092	8.00E-04	5.00E-04	0.09484	852575

rs595086	G	A		0.3163	-0.001	5.00E-04	0.03326	460826	0.6857	-0.0015	3.00E-04	3.14E-08	1157420	0.3087	-6.00E-04	5.00E-04	0.2363	809524
rs59646751	T	G		0.3088	-0.0057	5.00E-04	2.78E-30	460826	0.3043	-0.0024	3.00E-04	1.68E-19	1195880	0.3057	0.0054	5.00E-04	4.09E-26	852369
rs59860440	C	T		0.3263	-0.0013	5.00E-04	0.01138	460826	0.6874	-0.002	3.00E-04	1.24E-14	1159620	0.3108	0.001	5.00E-04	0.06266	810512
rs6029640	A	G		0.577	-0.0011	5.00E-04	0.02115	460826	0.5747	-0.0019	3.00E-04	4.99E-13	1017230	0.5774	5.00E-04	5.00E-04	0.389	670586
rs60551165	C	T		0.7697	-0.0029	5.00E-04	1.13E-07	460826	0.2736	-0.0017	3.00E-04	1.19E-09	1201600	0.723	0.0013	6.00E-04	0.02241	852349
rs6055748	A	G		0.6968	-0.0018	5.00E-04	0.000393	460826	0.6915	-0.0018	3.00E-04	7.28E-09	1035260	0.6952	0.0032	6.00E-04	1.65E-08	704303
rs60580012	T	C		0.334	-0.0017	5.00E-04	0.000681	460826	0.3216	-0.0018	3.00E-04	4.24E-12	1159620	0.3271	0.0014	5.00E-04	0.00723	810512
rs60691990	T	C		0.6639	-0.0018	5.00E-04	0.000315	460826	0.6715	-0.0016	3.00E-04	4.61E-08	1158870	0.6712	7.00E-04	6.00E-04	0.233	810011
rs60980181	A	T		0.139	-0.0018	7.00E-04	0.005679	460826	0.157	-0.0027	4.00E-04	2.90E-14	1198980	0.1581	-1.00E-04	7.00E-04	0.91	852671
rs60991551	A	C	TRUE	0.1314	-0.001	7.00E-04	0.159	460826	0.166	-0.0019	4.00E-04	4.93E-08	1201720	0.169	-4.00E-04	7.00E-04	0.5845	852467
rs61311941	A	G		0.9209	-0.0048	9.00E-04	4.43E-08	460826	0.8792	-0.0028	4.00E-04	5.61E-10	1142110	0.8716	6.00E-04	8.00E-04	0.4643	793206
rs61327861	A	G		0.1243	-0.0028	7.00E-04	5.57E-05	460826	0.1331	-0.0029	4.00E-04	1.70E-12	994592	0.1275	0.0033	8.00E-04	3.51E-05	661634
rs61482805	C	G	TRUE	0.6756	6.00E-04	5.00E-04	0.1907	456193	0.6824	-0.0021	3.00E-04	1.49E-15	1131240	0.6864	5.00E-04	5.00E-04	0.3349	795370
rs61546496	A	C		0.3353	-0.0022	5.00E-04	7.60E-06	459184	0.3267	-0.0016	3.00E-04	9.15E-10	1200900	0.3243	0.0014	5.00E-04	0.006561	851650
rs61830291	A	C	TRUE	0.9061	-0.0012	8.00E-04	0.1155	460826	0.9022	-0.004	5.00E-04	1.42E-17	993896	0.9029	-0.0011	9.00E-04	0.2315	662135
rs61910261	G	T		0.5721	-6.00E-04	5.00E-04	0.1943	460826	0.4106	-0.0014	3.00E-04	1.60E-08	1159620	0.5875	0.0027	5.00E-04	2.35E-07	810512
rs62035088	G	A		0.1681	-0.0035	6.00E-04	1.30E-08	459184	0.8358	-0.0027	3.00E-04	6.73E-16	1154210	0.1657	0.0024	7.00E-04	0.000433	810512
rs62071986	T	C		0.0393	-0.0046	0.0012	9.41E-05	460826	0.041	-0.0041	7.00E-04	6.25E-09	986333	0.0407	-0.0122	0.0014	8.08E-19	657961
rs62187537	C	T		0.0664	-0.0042	9.00E-04	5.81E-06	460826	0.9327	-0.0037	6.00E-04	2.07E-11	1039570	0.0678	0.0024	0.001	0.02142	703933
rs622076	A	G		0.1583	-0.0071	6.00E-04	3.74E-28	436765	0.1501	-0.004	4.00E-04	1.16E-25	1193290	0.1484	0.0016	7.00E-04	0.023	851473
rs62325228	G	T		0.1486	-0.002	6.00E-04	0.002313	460826	0.856	-0.0022	3.00E-04	3.33E-10	1201910	0.1496	4.00E-04	7.00E-04	0.5157	852665
rs62330280	A	G		0.515	-0.0034	5.00E-04	2.61E-13	436765	0.4959	-0.002	3.00E-04	1.97E-15	1201880	0.4923	0.0024	5.00E-04	7.16E-07	852628
rs62435145	T	G		0.6856	-0.0068	5.00E-04	3.12E-39	451330	0.6447	-0.0066	3.00E-04	8.01E-99	1057620	0.6315	0.0128	6.00E-04	2.19E-109	756284
rs62491533	T	C		0.8331	-0.0017	6.00E-04	0.004991	460826	0.8201	-0.0027	3.00E-04	3.87E-15	1200420	0.8226	0.0017	6.00E-04	0.009637	852623
rs62618693	C	T		0.044	-0.007	0.0011	2.47E-10	460826	0.9559	-0.0038	7.00E-04	2.51E-08	1019000	0.0446	0.0037	0.0013	0.004344	695717
rs6445924	G	A		0.1739	-0.0015	6.00E-04	0.01562	460826	0.8275	-0.0019	3.00E-04	1.05E-08	1039920	0.1737	8.00E-04	7.00E-04	0.2426	704285
rs6453319	C	A	TRUE	0.4532	-7.00E-04	5.00E-04	0.1557	460826	0.5741	-0.0014	3.00E-04	3.85E-08	973658	0.4266	7.00E-04	5.00E-04	0.1655	759969
rs6458868	T	C		0.6485	-0.0031	5.00E-04	1.91E-10	460826	0.6589	-0.002	3.00E-04	2.08E-14	1200420	0.6613	0.0037	5.00E-04	2.93E-12	852621
rs6468118	C	G		0.3849	-0.0013	5.00E-04	0.005686	460826	0.3713	-0.002	3.00E-04	2.92E-15	1200740	0.3661	0.0012	5.00E-04	0.0138	851502
rs6481598	G	C		0.7883	-0.0019	6.00E-04	0.000626	460826	0.2104	-0.0022	3.00E-04	3.42E-11	1201640	0.7934	4.00E-04	6.00E-04	0.4868	852387
rs6540119	T	A	TRUE	0.3256	-9.00E-04	5.00E-04	0.06234	460826	0.643	-0.0015	3.00E-04	3.20E-08	1153890	0.3542	8.00E-04	5.00E-04	0.1395	810512
rs6541410	C	G		0.6338	-0.002	5.00E-04	2.12E-05	460826	0.6258	-0.0015	3.00E-04	6.68E-09	1121560	0.6276	0.0014	5.00E-04	0.005472	810512
rs660931	C	T		0.2545	-0.0017	5.00E-04	0.001611	460826	0.6837	-0.0016	3.00E-04	5.67E-09	1197970	0.319	0.0017	6.00E-04	0.00245	848932
rs665731	T	C		0.1909	-0.0026	6.00E-04	1.60E-05	460826	0.1844	-0.0021	3.00E-04	1.36E-10	1174480	0.1823	0.002	7.00E-04	0.002641	830772
rs6700683	T	A		0.4719	-0.0019	5.00E-04	7.15E-05	436765	0.551	-0.0022	3.00E-04	1.61E-17	1115340	0.4466	0.0014	5.00E-04	0.005865	814907
rs6707326	G	A	TRUE	0.5657	0	5.00E-04	0.9285	460826	0.4469	-0.0016	3.00E-04	2.42E-10	1141710	0.5567	-0.0014	5.00E-04	0.01071	802942
rs6708702	A	G		0.294	-0.0017	5.00E-04	0.000813	460826	0.3531	-0.0019	3.00E-04	8.87E-13	1195810	0.3501	0.0028	5.00E-04	3.70E-07	851729
rs6779998	A	G	TRUE	0.5339	3.00E-04	5.00E-04	0.504	460826	0.5272	-0.0017	3.00E-04	6.20E-11	1196300	0.5251	-6.00E-04	5.00E-04	0.19	851729
rs67861508	G	A		0.6614	-0.0021	5.00E-04	2.08E-05	460826	0.3233	-0.0016	3.00E-04	2.30E-09	1158670	0.678	-4.00E-04	5.00E-04	0.4333	809561
rs688540	A	G		0.8638	-0.004	7.00E-04	2.36E-09	460825	0.8667	-0.0025	4.00E-04	1.95E-10	972754	0.8627	0.0014	8.00E-04	0.07666	646251
rs6968865	A	T		0.3778	-0.0015	5.00E-04	0.001983	460826	0.4043	-0.0023	3.00E-04	1.13E-19	1197150	0.4136	0.0031	5.00E-04	5.65E-10	852586
rs7005025	C	A		0.3672	-0.0022	5.00E-04	4.19E-06	460826	0.6423	-0.002	3.00E-04	5.01E-15	1158520	0.36	5.00E-04	5.00E-04	0.3378	809417
rs700753	G	C		0.3453	-0.0044	5.00E-04	5.13E-20	460826	0.6684	-0.0032	3.00E-04	1.09E-35	1201580	0.3331	0.0042	5.00E-04	3.00E-15	852330
rs7012637	G	A	TRUE	0.4773	-7.00E-04	5.00E-04	0.1409	460826	0.5118	-0.0027	3.00E-04	4.33E-26	1145310	0.4938	3.00E-04	5.00E-04	0.5372	801988
rs7017848	A	G		0.4058	-0.0012	5.00E-04	0.00926	460826	0.3932	-0.0014	3.00E-04	4.14E-08	1148190	0.3918	0	5.00E-04	0.986	804814
rs7027509	T	C	TRUE	0.3287	7.00E-04	5.00E-04	0.1724	460826	0.3243	-0.0016	3.00E-04	3.66E-10	1190590	0.3226	5.00E-04	5.00E-04	0.3542	852051
rs7086663	A	T		0.3961	-0.0011	5.00E-04	0.02266	460826	0.3821	-0.0014	3.00E-04	2.91E-08	1149260	0.3889	-3.00E-04	5.00E-04	0.5329	800153
rs7087356	T	A		0.5047	-0.0022	5.00E-04	1.23E-06	460826	0.4901	-0.0018	3.00E-04	2.92E-12	1159620	0.5091	0.0026	5.00E-04	2.56E-07	810512
rs7088058	C	T		0.4085	-0.0016	5.00E-04	0.000506	460826	0.6159	-0.0015	3.00E-04	3.17E-09	1200500	0.3828	3.00E-04	5.00E-04	0.5524	851263
rs7131509	C	T		0.6532	-0.0021	5.00E-04	1.15E-05	460826	0.3751	-0.0021	3.00E-04	1.91E-16	1159620	0.6139	0.0025	5.00E-04	1.72E-06	810512
rs71606723	T	A		0.7773	-0.0012	6.00E-04	0.02405	460826	0.2253	-0.0022	3.00E-04	6.48E-12	1201880	0.784	1.00E-04	6.00E-04	0.8687	852626
rs7161243	T	C	TRUE	0.7378	6.00E-04	5.00E-04	0.2635	460826	0.7177	-0.0016	3.00E-04	3.83E-09	1159620	0.7142	0	6.00E-04	0.9891	810512
rs7169629	G	C	TRUE	0.5164	-5.00E-04	5.00E-04	0.2528	460826	0.5015	-0.0019	3.00E-04	2.92E-14	1196050	0.4914	6.00E-04	5.00E-04	0.2485	852532
rs7178859	T	C		0.2093	-0.002	6.00E-04	0.000423	460826	0.2118	-0.0024	3.00E-04	2.36E-13	996528	0.2125	0.0016	7.00E-04	0.01282	661040
rs7196485	G	T		0.707	-0.0044	5.00E-04	1.62E-18	460826	0.2808	-0.0027	3.00E-04	6.20E-24	1200970	0.718	0.0039	5.00E-04	9.38E-13	851721
rs7199123	T	A		0.699	-0.0017	5.00E-04	0.000664	460826	0.3058	-0.0015	3.00E-04	2.31E-08	1195850	0.691	0	5.00E-04	0.9821	852339
rs7248248	A	T		0.4305	-0.0015	5.00E-04	0.000975	460826	0.4379	-0.0018	3.00E-04	1.30E-12	1017230	0.4343	7.00E-04	5.00E-04	0.216	670586
rs7259073	C	T		0.9288	-0.0048	9.00E-04	3.42E-07	456193	0.0859	-0.0038	6.00E-04	1.59E-10	896111	0.9304	0.0045	0.0011	5.83E-05	620251
rs72629024	G	C		0.8551	-0.0019	7.00E-04	0.003989	450809	0.2361	-0.0037	4.00E-04	2.93E-20	1045780	0.739	-0.0012	7.00E-04	0.102	757797
rs72683923	T	C		0.9808	0.0028	0.0017	0.09157	460826	0.9801	-0.0073	0.001	3.02E-13	961605	0.9802	0.0042	0.002	0.03364	641120
rs72801857	T	C		0.032	-0.0073	0.0013	2.17E-08	460826	0.0326	-0.0065	8.00E-04	1.05E-16	1046020	0.0335	0.0084	0.0015	9.53E-09	711582
rs72830437	A	G		0.0647	-0.001	9.00E-04	0.3079	460826	0.0695	-0.0028	5.00E-04	2.99E-08	1201660	0.0691	0.0024	0.001	0.01387	852416
rs72912510	A	G		0.1957	-0.0036	6.00E-04	5.59E-10	460826	0.1993	-0.0027	3.00E-04	3.36E-16	1157220	0.1919	0.0025	6.00E-04	0.000135	809561
rs73073442	C	T		0.0222	-0.0042	0.0016	0.007272	460826	0.9747	-0.0056	9.00E-04	5.24E-10	1026140	0.023	0.0082	0.0018	5.23E-06	697638
rs73077077	G	A		0.1843	-0.0015	6.00E-04	0.01093	460826	0.8076	-0.0021	3.00E-04	2.65E-10	1155670	0.1934	6.00E-04	7.00E-04	0.3716	807394
rs73117042	C	T		0.8628	-0.0015	7.00E-04	0.02962	460826	0.1332	-0.003	4.00E-04	5.68E-18	1201780	0.8655	0.0021	7.00E-04	0.003541	852526
rs7320064	G	A		0.8089	-0.0015	6.00E-04	0.009635	460826	0.1844	-0.002	3.00E-04	2.18E-09	1200970	0.8146	0.0023	6.00E-04	0.000258	851723
rs7324484	G	A		0.6519	-0.0032	5.00E-04	3.46E-11	460826	0.3517	-0.002	3.00E-04	4.41E-15	1196300	0.6464	0.002	5.00E-04	5.05E-05	851729
rs73245338	G	T		0.066	-0.0027	9.00E-04	0.003683	460826	0.9361	-0.0034	6.00E-04	1.09E-09	993101	0.0653	0.0022	0.0011	0.03822	662135
rs7326821	G	A		0.8259	-0.0048	6.00E-04	2.99E-15	460826	0.1731	-0.0021	3.00E-04	2.55E-10	1159620	0.8288	0.0034	7.00E-04	8.59E-07	810512
rs736820	A	G		0.3766	-0.0011	5.00E-04	0.02369	460826	0.3735	-0.0016	3.00E-04	2.18E-10	1157220	0.3746	2.00E-04	5.00E-04	0.7007	808106
rs73703112	T	G		0.6945	-0.0024	5.00E-04	1.98E-06	460826	0.6917	-0.0021	3.00E-04	1.97E-15	1201740	0.6964	0.0023	5.00E-04	2.69E-05	852494
rs7397189	T	G		0.7644	-0.0047	5.00E-04	5.62E-18	460826	0.7654	-0.0031	3.00E-04	9.44E-22	1062520	0.772	0.0065	6.00E-04	9.54E-25	768308
rs7405696	C	G		0.414	-0.003	5.00E-04	3.07E-10	456193	0.434	-0.0015	3.00E-04	1.42E-08	1142060	0.4271	-1.00E-04	5.00E-04	0.9134	800775
rs74331768	A	G		0.0985	-4.00E-04	8.00E-04	0.6177	453850	0.097	-0.003	5.00E-04	2.55E-09	924327	0.101	0.0029	9.00E-04	0.002123	621613
rs74748843	T	C		0.0232	-0.0033	0.0016	0.03429	460826	0.0545	-0.0046	7.00E-04	1.14E-09	1171780	0.0575	0.0071	0.0014	4.87E-07	826445
rs7475348	C	T		0.4892	-0.0018	5.00E-04	0.000129	460826	0.5241	-0.003	3.00E-04	5.44E-32	1159620	0.4681	2.00E-04	5.00E-04	0.6915	810512
rs7492724	G	A		0.711	-0.0026	5.00E-04	2.57E-07	460826	0.2796	-0.0022	3.00E-04	4.25E-16	1201910	0.7198	0.0026	5.00E-04	2.87E-06	852659
rs7514579	A	C		0.767	-0.0028	5.00E-04	3.02E-07	460826	0.7737	-0.0025	3.00E-04	7.12E-15	1159620	0.7761	0.0023	6.00E-04	9.94E-05	810512
rs75248620	G	A		0.082	-0.0016	8.00E-04	0.06327	460826	0.9188	-0.0036	5.00E-04	2.25E-13	1001420	0.0819	0.002	0.001	0.03945	662724
rs7535253	C	T		0.2125	-0.0021	6.00E-04	0.000265	460826	0.7596	-0.0024	3.00E-04	2.16E-13	1199770	0.2505	4.00E-04	6.00E-04	0.4697	850536
rs7536433	C	T		0.214	-0.0031	6.00E-04	3.01E-08	460826	0.7638	-0.0026	3.00E-04	2.11E-15	1196300	0.247	0.0016	6.00E-04	0.005167	851729
rs7539054	A	C		0.7337	-0.0026	5.00E-04	4.55E-07	460826	0.7097	-0.0021	3.00E-04	2.79E-14	1199260	0.6972	0.0019	5.00E-04	0.000402	852465
rs755865	G	A		0.1267	-0.0052	7.00E-04	7.60E-14	460826	0.8706	-0.0043	4.00E-04	1.41E-26	996438	0.1291	0.0081	8.00E-04	4.63E-24	662135
rs75625374	G	C	TRUE	0.0602	-0.0011	0.001	0.2538	460826	0.9403	-0.0036	6.00E-04	2.03E-10	1153620	0.0603	-0.0018	0.0011	0.09492	806359
rs7592697	T	C	TRUE	0.6568	1.00E-04	5.00E-04	0.8857	460826	0.6639	-0.0019	3.00E-04	9.75E-13	1159620	0.6648	6.00E-04	5.00E-04	0.2554	810512
rs76273615	G	A		0.8785	-0.0039	7.00E-04	2.18E-08	460826	0.1391	-0.0029	4.00E-04	4.07E-16	1201540	0.8611	0.0094	7.00E-04	3.29E-38	852291
rs7630893	T	C		0.7254	-0.0032	5.00E-04	4.80E-10	460826	0.722	-0.0045	3.00E-04	4.78E-63	1201810	0.723	4.00E-04	6.00E-04	0.4337	852562
rs7687209	C	T		0.4149	-0.0015	5.00E-04	0.001685	460826	0.5984	-0.0021	3.00E-04	5.44E-16	1158170	0.3995	5.00E-04	5.00E-04	0.3237	809060
rs77375846	C	T		0.87	-0.0024	7.00E-04	0.000434	460826	0.1314	-0.003	4.00E-04	5.55E-14	997635	0.8712	0.0014	8.00E-04	0.06606	662135
rs7740107	T	A		0.7408	-0.0016	5.00E-04	0.002221	460826	0.2621	-0.003	3.00E-04	1.99E-21	1200190	0.741	0.0013	6.00E-04	0.0271	852390
rs7766720	C	T		0.9175	-0.0035	9.00E-04	3.10E-05	460826	0.1035	-0.0026	4.00E-04	5.24E-09	1158180	0.8934	0.0055	9.00E-04	4.17E-10	810512
rs7769218	G	A		0.8066	-0.0031	6.00E-04	7.75E-08	460826	0.202	-0.002	3.00E-04	9.47E-10	1199370	0.7896	8.00E-04	6.00E-04	0.1515	851567
rs77897671	C	T		0.8301	-0.0026	6.00E-04	2.03E-05	460305	0.1864	-0.0022	3.00E-04	8.91E-11	1097010	0.8042	0.0015	6.00E-04	0.02076	781880
rs77924615	G	A		0.1958	-0.012	6.00E-04	6.29E-94	459184	0.8014	-0.0106	3.00E-04	8.53E-223	1158670	0.2023	0.0095	6.00E-04	1.17E-52	809561
rs7829427	A	G		0.2622	-0.0014	5.00E-04	0.009555	460826	0.2734	-0.0015	3.00E-04	2.72E-08	1191600	0.2742	0	6.00E-04	0.981	848251
rs78444298	A	G		0.0192	-0.0079	0.0017	2.55E-06	460826	0.0189	-0.012	0.001	2.19E-30	991164	0.0194	0.0055	0.002	0.005552	673168

rs784503	A	G		0.2316	-0.0014	5.00E-04	0.0103	460826	0.2376	-0.002	3.00E-04	6.73E-10	1200780	0.2297	9.00E-04	6.00E-04	0.1228	851530
rs792839	G	A	TRUE	0.3366	0	5.00E-04	0.9884	460826	0.677	-0.0016	3.00E-04	1.25E-09	1201750	0.3214	6.00E-04	5.00E-04	0.2247	852504
rs7947897	T	C		0.6027	-0.0016	5.00E-04	0.000448	460826	0.6173	-0.0014	3.00E-04	2.85E-08	1159620	0.619	0.0015	5.00E-04	0.004302	810512
rs795010	T	A	TRUE	0.7482	-4.00E-04	5.00E-04	0.3965	460826	0.2624	-0.0019	3.00E-04	1.33E-12	1201730	0.7331	-0.0014	6.00E-04	0.01206	852484
rs7963577	C	T		0.7738	-0.0026	5.00E-04	3.00E-06	460826	0.2153	-0.0022	3.00E-04	2.02E-11	1163680	0.7813	0.0028	6.00E-04	2.20E-06	852502
rs7983636	G	A		0.0782	-9.00E-04	9.00E-04	0.2756	460826	0.9221	-0.0031	5.00E-04	3.55E-10	1196300	0.0804	0.002	9.00E-04	0.02655	851729
rs79865452	A	G		0.9778	-0.0051	0.0016	0.001438	452343	0.9785	-0.0071	0.001	3.11E-12	891722	0.9781	0.0042	0.0019	0.02814	619862
rs80138475	C	T		0.1109	-0.0048	7.00E-04	4.20E-11	460826	0.8886	-0.0062	4.00E-04	8.64E-50	1047260	0.1119	0.0054	8.00E-04	4.05E-11	711621
rs80282103	T	A		0.915	-0.0087	8.00E-04	2.38E-25	460826	0.0868	-0.0081	5.00E-04	2.91E-62	1201760	0.9136	0.0066	9.00E-04	2.03E-14	852514
rs8058927	G	A		0.1473	-0.0042	7.00E-04	1.77E-10	460826	0.841	-0.0025	4.00E-04	1.12E-12	1190560	0.1607	0.0025	7.00E-04	0.000158	851729
rs8065496	T	C	TRUE	0.3853	7.00E-04	5.00E-04	0.1631	459184	0.3985	-0.0014	3.00E-04	4.29E-08	1150660	0.395	-0.001	5.00E-04	0.05259	844159
rs8073316	T	A		0.3019	-0.0021	5.00E-04	1.84E-05	460826	0.6823	-0.0015	3.00E-04	5.21E-09	1201840	0.3138	0.0019	5.00E-04	0.000262	852588
rs807624	G	T		0.3663	-0.0054	5.00E-04	4.74E-29	460826	0.6073	-0.0035	3.00E-04	4.69E-41	1192700	0.4104	0.0038	5.00E-04	5.86E-14	849346
rs8096658	G	C		0.5144	-0.0039	5.00E-04	1.25E-16	451330	0.4723	-0.0054	3.00E-04	1.88E-75	1059950	0.5359	0.0064	5.00E-04	1.10E-32	744965
rs8101667	C	T		0.3418	-0.005	5.00E-04	3.56E-25	460826	0.6316	-0.0047	3.00E-04	2.63E-73	1201920	0.3793	0.0021	5.00E-04	4.10E-05	852668
rs81205	C	A		0.5322	-0.0025	5.00E-04	6.47E-08	460826	0.4787	-0.003	3.00E-04	7.12E-32	1135770	0.5227	0.0019	5.00E-04	0.000298	792891
rs836968	C	T		0.2752	-0.004	5.00E-04	1.47E-14	460826	0.6813	-0.0018	3.00E-04	9.80E-12	1158670	0.3195	-0.0042	6.00E-04	7.65E-14	809561
rs848446	A	G		0.2842	-0.0044	5.00E-04	5.38E-18	460826	0.2713	-0.0041	3.00E-04	5.91E-54	1201720	0.2728	0.0037	5.00E-04	1.48E-11	852468
rs881858	A	G		0.6922	-0.0067	5.00E-04	1.54E-41	460826	0.7032	-0.0054	3.00E-04	5.10E-93	1195490	0.7078	-0.003	5.00E-04	4.56E-08	850920
rs883541	A	G		0.7681	2.00E-04	5.00E-04	0.7001	460825	0.7386	-0.0024	3.00E-04	6.17E-19	1191470	0.7399	0.0016	6.00E-04	0.005791	848128
rs925612	T	C	TRUE	0.482	-4.00E-04	5.00E-04	0.3714	460826	0.4953	-0.0014	3.00E-04	2.68E-08	1199560	0.4993	1.00E-04	5.00E-04	0.8942	851525
rs9318186	G	A	TRUE	0.4518	-4.00E-04	5.00E-04	0.4499	460826	0.5193	-0.0019	3.00E-04	4.72E-14	1195190	0.4846	-2.00E-04	5.00E-04	0.6776	850634
rs9376148	C	A		0.64	-0.0016	5.00E-04	0.0007	460826	0.3633	-0.0015	3.00E-04	3.36E-09	1014420	0.6343	7.00E-04	5.00E-04	0.1373	802037
rs9397738	G	A		0.8583	-0.0035	7.00E-04	1.18E-07	460826	0.1526	-0.003	4.00E-04	3.15E-17	1182520	0.8421	0.0033	7.00E-04	1.78E-06	834924
rs9465741	C	A		0.5408	-0.0027	5.00E-04	5.80E-09	460826	0.4843	-0.0021	3.00E-04	1.91E-16	1158670	0.5127	7.00E-04	5.00E-04	0.1587	809561
rs9480867	G	A		0.1574	-0.0025	6.00E-04	9.39E-05	460826	0.847	-0.0034	3.00E-04	9.81E-23	1199200	0.1542	0.0011	7.00E-04	0.1034	851400
rs948494	A	G		0.3382	-0.0055	5.00E-04	1.33E-29	460826	0.3357	-0.0034	3.00E-04	7.89E-40	1201890	0.3308	-0.0034	5.00E-04	2.08E-10	852643
rs950965	A	G		0.3479	-0.0033	5.00E-04	1.07E-11	460826	0.3808	-0.0018	3.00E-04	4.70E-12	1159620	0.3938	0.002	5.00E-04	8.84E-05	810512
rs9521719	A	G		0.3994	-0.0023	5.00E-04	6.46E-07	460826	0.3945	-0.0019	3.00E-04	4.79E-14	1195370	0.3959	0.001	5.00E-04	0.03438	851851
rs953492	A	G		0.4644	-0.0026	5.00E-04	1.39E-08	460826	0.4518	-0.0027	3.00E-04	1.09E-25	1196020	0.451	0.0018	5.00E-04	0.000167	852504
rs956006	C	T		0.3235	-0.0012	5.00E-04	0.01411	460826	0.6712	-0.0017	3.00E-04	1.10E-10	1158520	0.3244	8.00E-04	5.00E-04	0.1302	809417
rs9590675	G	T		0.3975	-0.0021	5.00E-04	7.96E-06	460826	0.5858	-0.0021	3.00E-04	1.72E-16	1196300	0.4137	0.0026	5.00E-04	1.87E-07	851729
rs963837	T	C		0.551	-0.0067	5.00E-04	1.09E-47	460826	0.5595	-0.0055	3.00E-04	9.85E-102	1154860	0.562	0.0087	5.00E-04	1.12E-64	806956
rs9649512	G	A		0.2895	-0.0041	5.00E-04	6.44E-16	460826	0.7117	-0.0025	3.00E-04	7.29E-16	898939	0.2895	-0.0016	6.00E-04	0.007735	619781
rs965484	C	T		0.4313	-0.0048	5.00E-04	1.28E-24	460826	0.5716	-0.0026	3.00E-04	7.41E-25	1201920	0.4238	0.0025	5.00E-04	2.73E-07	852666
rs9663482	C	G		0.1715	-0.0027	6.00E-04	1.51E-05	460826	0.1963	-0.0024	3.00E-04	2.77E-12	1201920	0.2032	0.0025	6.00E-04	3.01E-05	852675
rs9807214	A	G		0.2975	-0.0025	5.00E-04	8.45E-07	460826	0.2923	-0.0022	3.00E-04	2.73E-13	1201920	0.2896	-0.0042	5.00E-04	1.33E-14	852668
rs9812319	C	T		0.3388	-0.0041	5.00E-04	2.29E-17	460826	0.6652	-0.0022	3.00E-04	1.12E-17	1196300	0.337	0.0053	5.00E-04	2.93E-23	851729
rs9823161	G	A		0.6804	-0.0036	5.00E-04	4.95E-13	460826	0.3578	-0.0025	3.00E-04	3.52E-16	1120110	0.6287	0.0029	5.00E-04	6.85E-08	810512
rs9894634	T	C		0.5988	-0.0014	5.00E-04	0.002149	460826	0.5724	-0.0019	3.00E-04	3.66E-14	1200730	0.5622	0.0015	5.00E-04	0.003213	851493
rs9895661	C	T		0.8205	-0.0031	6.00E-04	2.78E-07	460826	0.2302	-0.0077	3.00E-04	2.51E-114	1191460	0.7515	-0.0019	6.00E-04	0.001361	848352
rs9932625	A	G		0.23	-0.0046	5.00E-04	2.76E-17	460826	0.2485	-0.0033	3.00E-04	1.15E-32	1201590	0.2468	0.0038	6.00E-04	4.63E-11	852345
rs9943067	T	C		0.4216	-0.0022	5.00E-04	2.21E-06	460826	0.4264	-0.0019	3.00E-04	3.76E-13	1060060	0.4284	0.0014	5.00E-04	0.006425	768291
rs9998449	G	A	TRUE	0.6983	-6.00E-04	5.00E-04	0.2069	460826	0.3274	-0.0017	3.00E-04	1.87E-10	1199440	0.6676	3.00E-04	5.00E-04	0.5516	851327

Supplemental Table 3. Localized averaged causal estimates calculated from 100 percentile ranges of strata according to the instrument-free exposure.

eGFR percentile	Age, sex, and 10 PCs adjusted								Age, sex, 10 PCs, and clinical covariates adjusted							
	eGFR (creatinine)				eGFR (cystatin C)				eGFR (creatinine)				eGFR (cystatin C)			
	OR	low 95% CI	high 95% CI	P value	OR	low 95% CI	high 95% CI	P value	OR	low 95% CI	high 95% CI	P value	OR	low 95% CI	high 95% CI	P value
1	0.960	0.914	1.009	0.107	0.994	0.961	1.029	0.745	0.940	0.878	1.007	0.077	0.973	0.927	1.022	0.273
2	0.967	0.910	1.029	0.290	0.958	0.921	0.997	0.033	1.048	0.964	1.140	0.268	0.997	0.943	1.053	0.902
3	0.981	0.916	1.050	0.580	0.972	0.931	1.015	0.202	0.894	0.814	0.983	0.021	0.948	0.891	1.008	0.086
4	0.943	0.879	1.012	0.105	1.016	0.969	1.064	0.520	1.009	0.914	1.115	0.852	1.025	0.955	1.100	0.496
5	0.990	0.920	1.065	0.787	0.952	0.909	0.998	0.040	0.957	0.873	1.049	0.348	0.994	0.932	1.060	0.856
6	1.017	0.947	1.091	0.649	0.967	0.921	1.016	0.184	1.012	0.921	1.111	0.811	0.915	0.855	0.981	0.012
7	0.962	0.896	1.034	0.295	0.992	0.945	1.041	0.734	0.969	0.870	1.080	0.573	1.013	0.946	1.085	0.706
8	0.936	0.866	1.012	0.098	0.933	0.885	0.984	0.010	0.918	0.833	1.013	0.088	0.986	0.916	1.061	0.706
9	1.063	0.987	1.146	0.108	1.010	0.958	1.065	0.704	1.021	0.920	1.132	0.699	0.928	0.864	0.996	0.040
10	0.960	0.885	1.043	0.334	0.999	0.950	1.052	0.982	0.998	0.904	1.100	0.960	1.077	0.994	1.167	0.071
11	1.059	0.982	1.143	0.137	0.973	0.923	1.025	0.305	1.020	0.919	1.131	0.713	0.941	0.868	1.019	0.134
12	0.991	0.914	1.075	0.833	1.025	0.972	1.081	0.363	0.998	0.901	1.105	0.969	1.063	0.985	1.147	0.117
13	0.966	0.881	1.059	0.457	0.998	0.946	1.052	0.932	1.075	0.962	1.202	0.203	0.993	0.920	1.072	0.865
14	0.993	0.917	1.075	0.862	0.968	0.918	1.020	0.222	1.053	0.951	1.166	0.318	1.023	0.946	1.106	0.572
15	0.975	0.903	1.053	0.521	0.985	0.932	1.042	0.603	0.922	0.831	1.024	0.128	0.955	0.888	1.028	0.220
16	1.010	0.935	1.092	0.800	1.008	0.957	1.061	0.765	1.148	1.018	1.293	0.024	1.005	0.934	1.081	0.890
17	0.998	0.917	1.085	0.957	1.022	0.968	1.079	0.431	0.918	0.823	1.023	0.122	1.119	1.034	1.211	0.005
18	0.948	0.875	1.027	0.191	1.002	0.951	1.057	0.936	0.961	0.861	1.073	0.480	1.024	0.949	1.105	0.537
19	1.037	0.951	1.131	0.414	0.986	0.933	1.042	0.612	0.890	0.791	1.001	0.053	0.998	0.927	1.075	0.962
20	0.978	0.900	1.062	0.595	1.048	0.989	1.110	0.115	0.900	0.810	0.999	0.048	1.013	0.937	1.095	0.745
21	0.956	0.885	1.032	0.246	1.054	0.989	1.124	0.108	1.032	0.924	1.153	0.578	1.003	0.921	1.091	0.950
22	0.986	0.907	1.072	0.740	0.977	0.922	1.036	0.445	0.960	0.864	1.065	0.439	1.012	0.934	1.096	0.771
23	0.999	0.922	1.081	0.972	1.029	0.970	1.092	0.342	0.904	0.809	1.012	0.079	1.050	0.975	1.131	0.199
24	0.979	0.901	1.064	0.620	0.985	0.932	1.040	0.576	1.082	0.973	1.202	0.145	1.018	0.944	1.098	0.642
25	1.002	0.917	1.094	0.970	0.964	0.910	1.021	0.209	0.918	0.827	1.019	0.107	0.937	0.869	1.009	0.085
26	1.103	1.015	1.199	0.021	0.966	0.914	1.021	0.222	0.975	0.880	1.080	0.629	0.991	0.919	1.069	0.821
27	1.019	0.940	1.104	0.652	0.968	0.910	1.029	0.295	0.993	0.885	1.114	0.903	0.970	0.891	1.057	0.487
28	0.981	0.897	1.073	0.674	0.988	0.934	1.045	0.676	1.143	1.032	1.265	0.010	0.958	0.886	1.036	0.279
29	0.950	0.873	1.035	0.241	0.962	0.907	1.020	0.193	0.967	0.856	1.093	0.594	1.027	0.948	1.112	0.511
30	0.998	0.919	1.084	0.966	0.995	0.937	1.056	0.868	1.104	0.983	1.241	0.096	1.007	0.931	1.088	0.870
31	0.968	0.886	1.058	0.475	1.046	0.990	1.105	0.110	1.009	0.902	1.128	0.880	1.009	0.936	1.088	0.816
32	0.947	0.871	1.029	0.200	1.056	0.992	1.125	0.089	1.020	0.914	1.137	0.725	0.998	0.928	1.074	0.964
33	0.973	0.898	1.054	0.502	1.011	0.952	1.073	0.730	1.022	0.908	1.151	0.717	1.032	0.957	1.114	0.415
34	1.018	0.933	1.110	0.686	1.061	0.995	1.132	0.070	1.032	0.935	1.140	0.530	1.007	0.926	1.094	0.874
35	1.058	0.982	1.141	0.140	1.020	0.960	1.083	0.530	0.994	0.899	1.098	0.900	1.007	0.912	1.111	0.897
36	1.003	0.923	1.091	0.936	0.930	0.877	0.986	0.015	0.982	0.882	1.094	0.739	1.016	0.939	1.098	0.697
37	1.057	0.966	1.156	0.228	1.006	0.953	1.062	0.818	0.962	0.862	1.073	0.487	0.972	0.901	1.049	0.463
38	0.959	0.883	1.042	0.324	0.942	0.886	1.002	0.056	0.976	0.884	1.078	0.628	0.975	0.893	1.066	0.582
39	1.013	0.932	1.101	0.766	1.020	0.959	1.085	0.529	1.011	0.916	1.115	0.833	0.968	0.885	1.058	0.468
40	0.976	0.902	1.056	0.543	1.022	0.958	1.091	0.501	1.001	0.904	1.109	0.981	0.965	0.897	1.039	0.349
41	0.975	0.901	1.055	0.525	0.997	0.934	1.065	0.929	0.953	0.858	1.057	0.360	0.919	0.846	0.999	0.048
42	1.000	0.924	1.083	0.997	1.016	0.960	1.076	0.578	1.011	0.906	1.130	0.840	1.025	0.943	1.115	0.558
43	0.980	0.908	1.058	0.605	0.998	0.938	1.061	0.938	1.023	0.920	1.138	0.675	0.989	0.908	1.078	0.798
44	1.037	0.953	1.127	0.401	0.994	0.935	1.056	0.840	1.031	0.933	1.139	0.552	0.995	0.914	1.082	0.900
45	0.950	0.879	1.027	0.198	1.016	0.957	1.079	0.603	1.009	0.898	1.134	0.877	0.932	0.851	1.020	0.124
46	0.960	0.885	1.041	0.325	0.970	0.909	1.034	0.343	0.943	0.850	1.046	0.269	0.968	0.888	1.054	0.454
47	1.007	0.937	1.083	0.847	1.011	0.946	1.082	0.739	1.028	0.928	1.139	0.598	0.983	0.903	1.069	0.686
48	0.987	0.909	1.072	0.760	0.945	0.887	1.007	0.080	1.011	0.905	1.128	0.852	1.012	0.917	1.116	0.815
49	1.014	0.936	1.099	0.727	1.046	0.976	1.120	0.206	1.058	0.947	1.182	0.318	1.044	0.958	1.137	0.325
50	0.916	0.844	0.994	0.034	1.030	0.968	1.096	0.349	1.010	0.906	1.127	0.853	0.988	0.908	1.075	0.780
51	1.019	0.934	1.111	0.674	1.071	1.000	1.146	0.050	1.015	0.914	1.128	0.777	0.995	0.909	1.090	0.918
52	1.062	0.974	1.158	0.174	0.979	0.918	1.044	0.515	0.987	0.875	1.114	0.836	1.014	0.930	1.107	0.748
53	1.003	0.919	1.096	0.940	1.002	0.939	1.069	0.952	1.001	0.895	1.121	0.979	1.071	0.975	1.176	0.151
54	0.962	0.883	1.048	0.375	0.975	0.914	1.040	0.436	0.980	0.876	1.096	0.719	1.069	0.982	1.163	0.124
55	1.007	0.925	1.097	0.866	0.984	0.920	1.053	0.646	0.980	0.856	1.123	0.773	0.985	0.898	1.081	0.750
56	0.972	0.883	1.071	0.569	1.033	0.964	1.107	0.356	0.940	0.831	1.064	0.328	1.088	1.003	1.179	0.041
57	1.058	0.972	1.152	0.194	1.018	0.949	1.091	0.622	1.066	0.944	1.204	0.300	1.041	0.951	1.140	0.384
58	0.979	0.887	1.081	0.680	0.927	0.867	0.992	0.027	1.006	0.899	1.126	0.912	0.965	0.875	1.065	0.478
59	1.025	0.922	1.139	0.646	1.044	0.977	1.115	0.203	0.969	0.854	1.099	0.623	1.014	0.926	1.109	0.770
60	0.925	0.839	1.020	0.120	1.002	0.937	1.071	0.962	0.945	0.828	1.078	0.397	0.952	0.867	1.046	0.307
61	0.956	0.871	1.050	0.349	1.007	0.940	1.079	0.839	1.203	1.040	1.393	0.013	1.024	0.918	1.142	0.676
62	1.140	1.032	1.260	0.010	0.962	0.896	1.033	0.287	1.104	0.968	1.259	0.140	0.919	0.834	1.013	0.088
63	1.017	0.919	1.126	0.738	0.980	0.909	1.056	0.593	1.078	0.958	1.212	0.211	0.969	0.885	1.060	0.487
64	1.019	0.923	1.126	0.705	0.969	0.900	1.043	0.395	1.037	0.922	1.168	0.543	1.023	0.936	1.118	0.614
65	1.101	1.000	1.213	0.050	0.973	0.898	1.055	0.512	1.159	1.018	1.320	0.026	1.062	0.969	1.164	0.195
66	1.012	0.915	1.119	0.819	0.936	0.865	1.013	0.102	0.960	0.850	1.084	0.509	1.050	0.953	1.156	0.325
67	1.008	0.909	1.118	0.884	1.018	0.948	1.093	0.623	1.058	0.937	1.194	0.361	0.948	0.864	1.041	0.264
68	0.982	0.892	1.081	0.706	0.958	0.887	1.035	0.278	1.028	0.906	1.168	0.667	1.010	0.922	1.105	0.836
69	1.137	1.028	1.257	0.013	1.006	0.936	1.081	0.881	1.040	0.918	1.178	0.536	0.979	0.890	1.076	0.655
70	1.072	0.970	1.185	0.171	0.955	0.886	1.030	0.235	1.091	0.955	1.246	0.200	0.965	0.864	1.077	0.523
71	1.028	0.933	1.132	0.577	0.977	0.906	1.053	0.541	0.916	0.804	1.045	0.192	1.028	0.940	1.124	0.542
72	1.027	0.920	1.146	0.639	0.971	0.900	1.047	0.440	0.952	0.834	1.087	0.469	1.035	0.947	1.130	0.450
73	0.995	0.897	1.103	0.919	1.008	0.928	1.094	0.858	0.955	0.840	1.086	0.483	1.018	0.928	1.118	0.700
74	1.005	0.907	1.114	0.922	0.997	0.925	1.074	0.930	0.922	0.812	1.047	0.209	0.973	0.886	1.069	0.571
75	1.040	0.942	1.148	0.436	1.056	0.984	1.133	0.133	1.050	0.926	1.190	0.444	1.088	0.985	1.201	0.096
76	1.057	0.958	1.167	0.269	1.005	0.921	1.096	0.917	1.068	0.946	1.206	0.286	1.010	0.910	1.121	0.854
77	1.108	1.008	1.219	0.033	1.045	0.961	1.136	0.303	1.107	0.982	1.248	0.097	0.968	0.886	1.058	0.475
78	1.018	0.926	1.120	0.708	0.975	0.906	1.050	0.506	1.075	0.956	1.209	0.226	1.061	0.957	1.177	0.262
79	1.015	0.919	1.121	0.766	0.905	0.829	0.988	0.026	1.021	0.904	1.153	0.737	0.945	0.862	1.037	0.231
80	1.061	0.961	1.170	0.240	1.027	0.953	1.108	0.481	1.014	0.902	1.140	0.817	0.989	0.900	1.086	0.814
81	1.050	0.959	1.149	0.290	1.011	0.938	1.091	0.770	1.064	0.942	1.203	0.317	1.071	0.964	1.189	0.202
82	1.109	1.011	1.216	0.028	1.035	0.963	1.113	0.351	0.985	0.869	1.116	0.812	1.030	0.928	1.143	0.577
83	1.011	0.922	1.108	0.820	1.005	0.927	1.089	0.912	1.017	0.905	1.143	0.781	1.050	0.953	1.158	0.324
84	1.030	0.934	1.135	0.557	1.086	1.010	1.167	0.025	1.052	0.943	1.175	0.363	0.962	0.882	1.050	0.388
85	0.947	0.861	1.040	0.256	1.051	0.976	1.132	0.186	1.084	0.964	1.219	0.178	1.060	0.968	1.160	0.207
86	0.973	0.886	1.068	0.565	0.996	0.928	1.069	0.911	0.978	0.875	1.093	0.696	1.084	0.975	1.207	0.137
87	0.931	0.853	1.017	0.112	0.973	0.900	1.052	0.491	1.020	0.916	1.137	0.714	0.951	0.868	1.042	0.285
88	1.047	0.955	1.147	0.328	1.077	1.002	1.157	0.044	1.004	0.897	1.125	0.941	1.050	0.958	1.150	0.299
89	0.942	0.858	1.035	0.212	1.067	0.994	1.146	0.072	0.944	0.840	1.062	0.338	1.017	0.933	1.108	0.702
90	0.968	0.878	1.068	0.515	1.011	0.938	1.091	0.771	1.015	0.907	1.136	0.796	1.039	0.955	1.131	0.374
91	0.938	0.860	1.024	0.152	1.013	0.937	1.095	0.755	0.995	0.892	1.110	0.927	1.036	0.947	1.133	0.442
92	1.054	0.964	1.153	0.246	0.985	0.917	1.059	0.684	1.034	0.932	1.146	0.532	1.064	0.977	1.160	0.156
93	1.024	0.938	1.117	0.599	1.050	0.976	1.130	0.192	0.992	0.882	1.117	0.899	0.963	0.890	1.041	0.340
94	1.182	1.070	1.307	0.001	1.055	0.977	1.139	0.175	1.021	0.929	1.123	0.663	1.023	0.942	1.112	0.589
95	1.016	0.926	1.116	0.734	0.955	0.893	1.022	0.186	0.952	0.861	1.053	0.339	1.066	0.985	1.153	0.112
96	0.925	0.851	1.005	0.066	1.049	0.972	1.132	0.219	0.993	0.902	1.092	0.882	1.021	0.940	1.109	0.619
97	1.032	0.953	1.118	0.432	1.030	0.967	1.098	0.360	1.108	1.001	1.227	0.048	1.014	0.928	1.108	0.755
98	0.969	0.886	1.061	0.498	0.959	0.894	1.029	0.242	1.005	0.918	1.100	0.914	0.982	0.913	1.056	0.631
99	1.128	1.040	1.223	0.004	0.967	0.904	1.034	0.329	0.904	0.822	0.993	0.035	1.027	0.961	1.096	0.433
100	1.035	0.967	1.108	0.326	1.018	0.961	1.079	0.536	1.011	0.939	1.088	0.768	1.000	0.943	1.060	0.995

Localized averaged causal effect was calculated in 100 strata of the population. The meta-regression results by the fractional polynomial method are presented in Table 2.

The unit of the localized averaged causal effect was odds for risks of myocardial infarction for an exponential increase in eGFR.

Clinical covariates included in the adjusted model were body mass index, systolic blood pressure, hypertension medication history, diabetes mellitus diagnosis, hemoglobin A1c, medication history for dyslipidemia, triglycerides, high-density lipoprotein and low-density lipoprotein cholesterols, and urine microalbumin levels.
